# Identification of CCCH Zinc Finger Proteins Family in Moso Bamboo (*Phyllostachys edulis*), and *PeC3H74* Confers Drought Tolerance to Transgenic Plants

**DOI:** 10.3389/fpls.2020.579255

**Published:** 2020-11-09

**Authors:** Feng Chen, Huan-Long Liu, Kang Wang, Ya-Meng Gao, Min Wu, Yan Xiang

**Affiliations:** ^1^Laboratory of Modern Biotechnology, School of Forestry and Landscape Architecture, Anhui Agricultural University, Hefei, China; ^2^National Engineering Laboratory of Crop Stress Resistance Breeding, Anhui Agricultural University, Hefei, China

**Keywords:** CCCH zinc finger proteins, moso bamboo (*Phyllostachys edulis*), *PeC3H74*, drought tolerance, stomatal

## Abstract

CCCH zinc finger proteins are a class of important zinc-finger transcription factors and have functions in various plant growth and stress responses, but their functions in moso bamboo (*Phyllostachys edulis*) are unclear. In this current study, we main investigated the structures, phylogenetic relationships, promoter elements and microsynteny of *PeC3Hs*. In this research, 119 CCCH zinc finger proteins (*PeC3H1-119*) identified genes in moso bamboo were divided into 13 subfamilies (A-M) based on phylogenetic analysis. Meanwhile, moso bamboo were treated with abscisic acid (ABA), methyl jasmonate (Me-JA) and gibberellic acid (GA) and 12 CCCH genes expression levels were assayed using qRT-PCR. In the three hormone treatments, 12 genes were up-regulated or down-regulated, respectively. In addition, *PeC3H74* was localized on the cytomembrane, and it had self-activation activities. Phenotypic and physiological analysis showed that *PeC3H74* (*PeC3H74-OE*) conferred drought tolerance of transgenic *Arabidopsis*, including H_2_O_2_ content, survival rate, electrolyte leakage as well as malondialdehyde content. Additionally, compared with wild-type plants, transgenic *Arabidopsis thaliana* seedling roots growth developed better under 10 μM ABA; Moreover, the stomatal of over-expressing *PeC3H74* in *Arabidopsis* changed significantly under ABA treatment. The above results suggest that *PeC3H74* was quickly screened by bioinformatics, and it may enhanced drought tolerance in plants through the ABA-dependent signaling pathway.

## Introduction

Moso bamboo is one of the most important forest types in China. Moso bamboo plays an important role not only in the ecological environment but also in China’s rural economy (Zhang X. P. et al., 2019). Transcription factors (TFs) not only regulate plant growth and development but also regulate the biosynthesis of plant secondary metabolites (Zhang et al., 2009). Zinc finger transcription factors, as one of the largest transcription factor (TF) families in plants, play a role in many biological processes, such as morphogenesis (Stege et al., 2002), signal transduction and environmental stress responses (Takatsuji, 1998). Nowadays, many zinc finger proteins have been found in plants, such as CCCH-type (Yang et al., 2020), ERF (Nakano et al., 2006), WRKY (Cheng et al., 2020), and DOF (Lijavetzky et al., 2003).

CCCH zinc finger protein contains a typical C3H-type motif (three cysteines and one histidine), and form a class of proteins that mainly exist in diverse eukaryotic organisms (Blackshear, 2002). So far, CCCH zinc finger family genes have been identified in many organisms, such as humans (55) (Liang et al., 2008), mouse (58) (Liang et al., 2008), *Arabidopsis thaliana* (68) (Wang D. et al., 2008), rice (*Oryza sativa*) (67) (Wang D. et al., 2008), poplar (*Populus trichocarpa*) (91) (Chai et al., 2012), *Brassica rapa* (103) (Pi et al., 2018), and tomato (*Solanum lycopersicum*) (80) (Xu, 2014). In rice and *A. thaliana*, based on the specially ordered cysteines and histidine in the CCCH domain, the sequence of the motif was determined to be C-X_4__–__15_-C-X_4__–__6_-C-X_3_-H (X for any amino acid) and CCCH protein contains at least one, and at most six, CCCH zinc finger domains (Wang D. et al., 2008). In maize, the sequence for these motifs was defined as C-X_4__–__17_-C-X_4__–__6_-C-X_3_-H, meanwhile, *ZmC3H17* contains 7 CCCH zinc finger domains (Peng et al., 2012). In many plants, C-X_7__/__8_-C-X_5_-C-X_3_-H is the most common domain (Pi et al., 2018).

CCCH zinc finger proteins are involved in developmental processes in plants, including seed germination (Kim et al., 2008), flowering and senescence (Yan et al., 2017), embryo development (Li and Thomas, 1998), and secondary wall synthesis (Zhang et al., 2018). For example, KHZ1 (*AtC3H36*) and KHZ2 (*AtC3H52*) participate in flowering and regulating leaf senescence in *A. thaliana* (Yan et al., 2017). In rice, *OsTZF1* regulates leaf-delayed senescence by regulating stress-response genes (Jan et al., 2013). In poplar, *PdC3H17* and *PdC3H18* positively regulate secondary wall formation (Chai et al., 2014). *CCCH* genes not only contribute to plant developmental processes, they also regulate biotic and abiotic stress responses. In *A. thaliana*, *AtC3H17*, a non-tandem zinc finger gene, positively regulates salt tolerance through an abscisic acid (ABA)-dependent pathway (Seok et al., 2018). In addition, *AtTZF4*, *5* and *6* play important roles in seed germination by regulating ABA, light and gibberellic acid (GA) (Bogamuwa and Jang, 2013). *IbC3H18*, which is a nuclear transcriptional activator, regulates the expression of abiotic stress response genes associated with ABA signaling (Zhang H. et al., 2019).

CCCH proteins have been studied in *A. thaliana*, rice, poplar and maize (*Zea mays* L.) (Chen et al., 2017). A previous study showed that CCCH plays a role in growth and responses to various stresses (Yan et al., 2017). However, they have not been studied in moso bamboo. In the current study, we investigated the structures, phylogenetic relationships, promoter elements, scaffold locations and microsynteny of 119 identified *PeC3Hs*. Meanwhile, we investigated 12 genes responses to ABA, Me-JA and GA treatments. In addition, the subcellular localizations and transcriptional activities of *PeC3H74* were analyzed. Then, this gene was over expressed in *Arabidopsis*. *PeC3H74* promoted root growth under ABA condition at seedling stages in transgenic *Arabidopsis*. *PeC3H74* increased the drought-stress tolerance and decreased the ABA sensitivity of transgenic plants. Thus, rapid screening of bioinformatics to *PeC3H74*, and it might participated in the positive regulation of drought-stress responses through an ABA-dependent pathway in plants.

## Materials and Methods

### *CCCH* Gene Identification

The GIGADB Dateset^1^ provided the basic data, including protein numbers, cDNA sequence numbers and open reading frame lengths, as well as the genomic sequences and their scaffold positions. The published rice CCCH proteins were originally used as probes to search for CCCH members in the moso bamboo genome with BLASTP and TBLASTN having an E-value cutoff set as 1e-005, and the sequences were detected again with SMART^2^ (Letunic et al., 2004) and Pfam^3^ (Finn et al., 2008) to ensure the existence of the CCCH domain. ExPASY provided information about molecular weights and isoelectric points (Gasteiger et al., 2003).

### Phylogenetic Analysis and Gene Structure

The ClustalX (version 1.83) program (Chai et al., 2012) was used for the multiple sequence alignment of CCCH sequences of moso bamboo. The neighbor-joining method of MEGA 6.0 software^4^ was used to construct the phylogenetic trees. Bootstrapping of each branch was performed using 1,000 replications. MEGA 6.0 and ClustalX programs were used to construct the integrated phylogenetic tree of moso bamboo, maize and rice. The Gene Structure Display Server program (Han et al., 2014) was used to study the intron/exon structure of *PeC3H* genes.

The MEME^5^ program was used to determine the conserved motifs of the 119 amino acid sequences of the *PeC3H* genes with the following parameters: the maximum number of motifs was 15 and the optimum width was 18–200 residues (Ma et al., 2014). In addition, sequence logos of the CCCH zinc finger motifs were produced using online WebLogo software (Crooks et al., 2004).

### Gene Duplication and *K*_a_/*K*_s_ Analysis

The genomic location of *PeC3H* genes were identified according to the information provided by moso Bamboo SMRT database, and the collinearity of CCCH gene family of bamboo were analyzed by using the default parameters of MCScanX (Wang et al., 2012) and shown by Circos (Krzywinski et al., 2009). To study relationships between orthologous *PeC3H* genes and other selected species, the homolinear analysis maps were constructed using Dual Synteny Plotter software^6^. DnaSP 5 software was used to calculate the PeC3H’s *K*_a_ and *K*_s_ values. The duplication time (*T*) was calculated as *T* = *K*_s_/2λ × 10^–6^ million years ago (MYA) (λ = 6.5 × 10^–9^ for grasses) (Gaut et al., 1996).

### *Cis*-element Analysis

To study the *cis*-elements of *PeC3H* genes’ promoter sequences, the *cis*-elements upstream of the transcription initiation site (2,000 bp of genomic DNA sequence) were analyzed using the PlantCARE program (Liu et al., 2009).

### Plant Materials and Stress Treatments

Bamboo seedlings gathered from Guilin City, Guangxi Province, China and were cultured in an artificial climate incubator for 7 weeks (26 ± 2°C, 16 h light) in preparation for subsequent experiments. Sow the moso bamboo seeds in a round pot (diameter 20.5 cm, height 19 cm). Afterward, the seedlings were sprayed independently with H_2_O, 100-μM ABA, 100-μM Me-JA, and 100-μM GA solutions (10 ml) at 0 h. Untreated seedlings served as the controls. Leaf samples were taken at different times (1, 3, 6, 12, and 24 h). Subsequently, total RNAs of these samples were extracted using the TRIzol method. RNA was reversed transcribed into cDNA using a PrimeScript^®^ RT Reagent Kit (TaKaRa, Dalin, China).

### Quantitative Real-Time PCR (qRT-PCR)

To study the expression levels of *PeC3H*s under different hormone-treatment conditions, 12 genes were selected for qRT-PCR analysis. The specific primers for the 12 selected *PeC3H* genes were designed using Primer 5.0 software, and the intrinsic membrane protein 41 (TIP41) was used as the reference gene (Fan et al., 2013). The program settings for the qRT-PCR were as follows: 95°C for 30 s; 40 cycles of 95°C for 5 s, 55–60°C for 10 s, and 72°C for 10 s. The relative expression levels of relevant genes were calculated using the following formula: 2^–ΔΔ^*^*C*^*^*T*^ [ΔCT = *C*_T_, _Target_ − *C*_T_,_TIP__41_. ΔΔ*C*_T_ = Δ*C*_T_ − Δ*C*_T_, _H__2__O_]. (Schmittgen and Livak, 2008). GraphPad5 was used to analyze the data.

### Subcellular Localization and Transactivation Activity

The full-length coding sequence of *PeC3H74* was obtained using specific primers (F: ATGAC TCCCTTGACTGGTTTCTTGACTGTT, R: GATAAGCAGA TTGGTAGCCTAGCA ACATAG) and cloned into pCAMBIA1305, containing CAMV35S and green fluorescent protein (GFP), using *Xba*I and *Sam* I restriction sites. PeC3H74-GFP was introduced into *Agrobacterium tumefaciens* EHA105, then injected into *Nicotiana tabacum* and observed using a confocal laser scanning microscope (Carl Zeiss LSM710, Germany) (Zhou et al., 2013).

The pGBKT7 vector (Clontech, Palo Alto, CA, United States) was used to study the transcriptional activity of the PeC3H74 protein in yeast. The full-length PeC3H74 was PCR amplified using gene-specific primers (F: GGAATTCATGACTCCCTTGACTGGTTT; R: ACGCGTCGACGATAAGCAGATTGGTAGCCT) and cloned into pGBKT7 vector containing a GAL4 DNA-binding domain. The empty plasmids (pGBKT7) and pGBKT7-53 + pGADT7-T antigen as negative and positive experimental controls. pGBKT7-PeC3H74, pGBKT7 and pGBKT7-53 + pGADT7-T were transformed in yeast strain by the lithium acetate method, respectively. The transformants were dropped on various SD selective media, namely SD/–Trp and SD/–Trp/–His/–Ade/X-α-GAL and incubated at 30°C for 3–5 days.

### Phenotypic Analysis of Transgenic Plants

To determine drought tolerance, 3-week-old plants (OE-6, OE-7, OE-9, WT) were withholding water for a week. The electrolyte leakage was measured according to previous studies (McKersie et al., 1996). MDA and H_2_O_2_ content was measured using Biochemical Assay Kit (BC0025 and BC3595, Liandong U Valley A85, Tongzhou District, Beijing, China). According to the method provided by (Kumar et al., 2014), DAB staining of the leaves after drought treatment. *PeC3H74-OE* transgenic *Arabidopsis* seedlings were grown on 1/2 MS medium for 3 days, and then transferred to 1/2 MS medium containing 10 μM ABA for vertical cultivation for 7 days, and the change in root length was observed.

### ABA Sensitivity Test

The mature *Arabidopsis* leaves were soaked in (0 and 1 μM) ABA for 6 h, and then transferred to a solution containing 25% propanetriol and 2 g/mL chloral hydrate for 4 days. After removing chlorophyll, observe and measure under a fluorescence microscope. The stomata ratios of width to length were >0.5 (open), 0.5–0.2 (partially closed) and <0.2 (closed), respectively.

### Statistical Analysis

Statistical significance was determined using a paired Student’ s *t*-test^9^. The mean ± standard error from the mean (SE) of at least three replicates are presented, and significant differences relative to controls are indicated at ^∗^*p* < 0.05 and ^∗∗^*p* < 0.01.

## Results

### Identification of the *PeC3H* Genes

Through a BLASTP preliminary screening, 123 putative CCCH-encoding genes of moso bamboo were obtained. The present of the CCCH domain was determined by Pfam and SMART, and 119 potential CCCH sequences were ultimately identified as *CCCH* genes (*PeC3H1-119*) (Liu et al., 2011). Detailed information of *PeC3H* genes are given in Table 1. The identified *PeC3H* genes encoded proteins ranging from 73 to 2,039 amino acids, with a mean length of 533 amino acids. The greatest and lowest molecular weights were 224.86 and 8.12 kDa, respectively.

**TABLE 1:** Detailed information on conserved amino acid sequences and motif lengths.

Name	Gene ID	Location	CDS length (bp)	Protein			Exons	Number of CCCH motif
				Size (aa)	MW (d)	pI		
PeC3H1	PH02Gene00351	Locus = hic_scaffold_24:69453362:69457139: −	2139	713	85994.7	5.39	3	3
PeC3H2	PH02Gene00385	Locus = hic_scaffold_24:68911642:68914988: +	933	311	32112.29	9.43	3	3
PeC3H3	PH02Gene00402	Locus = hic_scaffold_24:68615392:68623684: −	1326	442	47633.8	6.53	4	1
PeC3H4	PH02Gene01488	Locus = hic_scaffold_3:82184822:82192897: +	1566	522	55806.15	6.28	12	3
PeC3H5	PH02Gene02119	Locus = hic_scaffold_20:45960082:45967116: −	1317	439	50513.03	9.08	11	1
PeC3H6	PH02Gene02344	Locus = hic_scaffold_7:37901263:37901850: +	507	169	18214.9	6.48	2	1
PeC3H7	PH02Gene02576	Locus = hic_scaffold_18:17302347:17304766: −	675	225	24176.96	7.54	2	3
PeC3H8	PH02Gene03339	Locus = hic_scaffold_17:22555763:22561710: −	1302	434	46492.74	7.87	9	2
PeC3H9	PH02Gene04182	Locus = hic_scaffold_9:63015691:63016775: +	999	333	36065.58	8.39	2	2
PeC3H10	PH02Gene04254	Locus = hic_scaffold_9:61562372:61563223: +	852	284	32665.12	9.41	1	2
PeC3H11	PH02Gene04361	Locus = hic_scaffold_18:2965068:2966873: +	1806	602	63686.28	8.28	1	2
PeC3H12	PH02Gene04626	Locus = hic_scaffold_18:31829128:31830078: −	864	288	33843.49	8.97	2	2
PeC3H13	PH02Gene04944	Locus = hic_scaffold_16:20764288:20768323: −	1101	367	41914.83	5.22	8	2
PeC3H14	PH02Gene05077	Locus = hic_scaffold_18:23743915:23745252: +	1173	391	42430.31	8.59	3	2
PeC3H15	PH02Gene05151	Locus = hic_scaffold_18:25711501:25719943: −	5694	1898	204615.05	4.61	12	1
PeC3H16	PH02Gene05204	Locus = hic_scaffold_23:36972822:36976625: +	492	164	19141.81	9.15	5	3
PeC3H17	PH02Gene05739	Locus = hic_scaffold_16:8567617:8576807: +	1302	434	47084.13	8.68	7	5
PeC3H18	PH02Gene06226	Locus = hic_scaffold_3:54427883:54433247: +	2301	767	87923.88	8.5	9	2
PeC3H19	PH02Gene06968	Locus = hic_scaffold_10:10793499:10795505: −	2007	669	71258.13	6.2	1	2
PeC3H20	PH02Gene08040	Locus = hic_scaffold_7:42187438:42188214: −	495	165	18092.3	5.68	2	1
PeC3H21	PH02Gene08203	Locus = hic_scaffold_18:9338708:9340041: −	1188	396	41881.77	6.47	2	2
PeC3H22	PH02Gene08432	Locus = hic_scaffold_11:23303114:23318103: −	1326	442	47523.4	8.69	6	5
PeC3H23	PH02Gene08811	Locus = hic_scaffold_6:1670330:1673276: +	498	166	17946.09	8.88	5	1
PeC3H24	PH02Gene09684	Locus = hic_scaffold_24:7737820:7745991: −	2265	755	82957.27	6.34	6	1
PeC3H25	PH02Gene09722	Locus = hic_scaffold_24:8931972:8941394: −	1023	341	38469.89	8.47	9	1
PeC3H26	PH02Gene10304	Locus = hic_scaffold_17:87548005:87550456: −	900	300	31344.5	9.51	3	3
PeC3H27	PH02Gene10635	Locus = hic_scaffold_14:98422946:98423763: +	606	202	21840.35	8.83	3	2
PeC3H28	PH02Gene11220	Locus = hic_scaffold_8:63804613:63812546: +	5487	1829	200396.22	7.09	9	5
PeC3H29	PH02Gene12613	Locus = hic_scaffold_3:11732446:11733297: −	852	284	32972.38	9.07	1	2
PeC3H30	PH02Gene12713	Locus = hic_scaffold_8:8132158:8135447: −	1452	484	54138.01	7.49	4	1
PeC3H31	PH02Gene12814	Locus = hic_scaffold_19:27214307:27217076: −	2055	685	72639.82	6.23	8	1
PeC3H32	PH02Gene13318	Locus = hic_scaffold_4:12751633:12755326: +	1527	509	57630.66	8.17	7	1
PeC3H33	PH02Gene13668	Locus = hic_scaffold_6:37462284:37471005: −	6117	2039	224855.33	7.55	8	5
PeC3H34	PH02Gene14940	Locus = hic_scaffold_16:110649779:110650918: +	1140	380	40455.41	6.67	1	2
PeC3H35	PH02Gene15731	Locus = hic_scaffold_22:54826067:54829550: −	2865	955	104892.94	9.03	3	3
PeC3H36	PH02Gene16079	Locus = hic_scaffold_24:28791604:28806827: −	2826	942	101166.88	8.74	4	1
PeC3H37	PH02Gene16813	Locus = hic_scaffold_17:16365111:16369106: +	588	196	21745.12	9.54	8	1
PeC3H38	PH02Gene17017	Locus = hic_scaffold_14:103249981:103256751: −	1146	382	41782.09	9.33	3	2
PeC3H39	PH02Gene17257	Locus = hic_scaffold_6:11538169:11541393: −	1452	484	54078.99	7.86	4	1
PeC3H40	PH02Gene17992	Locus = hic_scaffold_4:1596517:1599302: +	2043	681	72282.53	6.21	8	1
PeC3H41	PH02Gene18149	Locus = hic_scaffold_8:32501708:32505864: +	1107	369	41699.47	8.2	1	4
PeC3H42	PH02Gene18259	Locus = hic_scaffold_21:25219541:25221778: −	2238	746	79153.14	6.12	1	2
PeC3H43	PH02Gene18357	Locus = hic_scaffold_17:4461067:4464481: +	1515	505	55752.14	5.33	4	1
PeC3H44	PH02Gene18565	Locus = hic_scaffold_3:61717869:61718968: +	732	244	27261.12	9.67	1	3
PeC3H45	PH02Gene19288	Locus = hic_scaffold_15:45871444:45874350: −	2013	671	73883.02	6	8	1
PeC3H46	PH02Gene19939	Locus = hic_scaffold_3:100319888:100325264: +	480	160	18259.63	8.87	5	1
PeC3H47	PH02Gene19983	Locus = hic_scaffold_14:39188544:39196040: −	1557	519	56892.48	8.57	12	6
PeC3H48	PH02Gene20573	Locus = hic_scaffold_23:3606988:3610056: −	921	307	31738.75	9.49	3	3
PeC3H49	PH02Gene22177	Locus = hic_scaffold_14:33650525:33654318: +	2145	715	77659.5	5.62	7	3
PeC3H50	PH02Gene22259	Locus = hic_scaffold_7:49246893:49248010: +	1029	343	37002.87	8.7	2	1
PeC3H51	PH02Gene22705	Locus = hic_scaffold_15:2657233:2667145: +	5124	1708	187416.76	5.86	12	1
PeC3H52	PH02Gene23823	Locus = hic_scaffold_14:68435709:68442162: +	1110	370	42198.11	5.13	8	2
PeC3H53	PH02Gene23886	Locus = hic_scaffold_13:56435451:56437253: −	378	126	14378.42	9.38	2	1
PeC3H54	PH02Gene24845	Locus = hic_scaffold_9:56938085:56938867: −	573	191	21300.2	8.16	1	2
PeC3H55	PH02Gene24944	Locus = hic_scaffold_9:3656080:3657879: +	1488	496	52702.87	8.64	5	2
PeC3H56	PH02Gene25228	Locus = hic_scaffold_6:39239720:39242561: +	903	301	31301.64	9.62	2	3
PeC3H57	PH02Gene26317	Locus = hic_scaffold_11:19983230:19997957: −	1542	514	55772.62	8.03	11	5
PeC3H58	PH02Gene26949	Locus = hic_scaffold_16:56532184:56557093: +	1443	481	52242.12	6.73	12	6
PeC3H59	PH02Gene27145	Locus = hic_scaffold_6:29949382:29957623: −	1968	656	71754.83	6.46	7	3
PeC3H60	PH02Gene27190	Locus = hic_scaffold_5:30938287:30942416: +	1881	627	74055.7	9.84	10	1
PeC3H61	PH02Gene27333	Locus = hic_scaffold_13:22925943:22929622: −	708	236	25130.78	9.51	8	2
PeC3H62	PH02Gene27671	Locus = hic_scaffold_11:30998901:31001429: +	1809	603	63791.94	5.74	2	2
PeC3H63	PH02Gene27920	Locus = hic_scaffold_3:103337471:103341054: +	1959	653	71780.8	5.39	3	3
PeC3H64	PH02Gene28052	Locus = hic_scaffold_17:268540:272051: −	1959	653	71780.8	5.39	3	3
PeC3H65	PH02Gene28235	Locus = hic_scaffold_13:82402496:82404242: +	633	211	23896.62	9.1	2	4
PeC3H66	PH02Gene29104	Locus = hic_scaffold_16:113779419:113785985: +	1074	358	39559.37	6.07	7	2
PeC3H67	PH02Gene29538	Locus = hic_scaffold_5:50744746:50745053: +	219	73	8191.12	6.01	2	1
PeC3H68	PH02Gene29764	Locus = hic_scaffold_6:70803072:70812489: +	1026	342	43558.27	8.61	10	1
PeC3H69	PH02Gene30888	Locus = hic_scaffold_13:31203042:31216767: −	1332	444	47739.82	8.7	7	5
PeC3H70	PH02Gene32013	Locus = hic_scaffold_16:51432126:51433247: +	1026	342	36803.46	8.92	2	2
PeC3H71	PH02Gene32078	Locus = hic_scaffold_22:59516932:59519801: −	1791	597	63647.33	6.11	8	1
PeC3H72	PH02Gene32291	Locus = hic_scaffold_21:42390504:42391729: −	1074	358	38558.81	8.5	2	1
PeC3H73	PH02Gene33170	Locus = hic_scaffold_15:98643212:98645947: +	939	313	36281.83	9.21	4	1
PeC3H74	PH02Gene33725	Locus = hic_scaffold_15:81676052:81678364: +	2313	771	81735.37	6.81	1	2
PeC3H75	PH02Gene34123	Locus = hic_scaffold_20:39387478:39389025: −	903	301	31619.24	9.55	2	3
PeC3H76	PH02Gene34597	Locus = hic_scaffold_14:55686484:55687233: +	426	142	15979.11	8.97	2	1
PeC3H77	PH02Gene34666	Locus = hic_scaffold_17:93580969:93587051: −	1311	437	49829.27	9.19	11	1
PeC3H78	PH02Gene34953	Locus = hic_scaffold_2:10801492:10811155: +	1362	454	48959.59	9.09	8	5
PeC3H79	PH02Gene36258	Locus = hic_scaffold_24:1644860:1685511: +	2661	887	99389.51	5.38	12	3
PeC3H80	PH02Gene36261	Locus = hic_scaffold_24:1965350:1967038: +	591	197	22257.76	8.02	2	3
PeC3H81	PH02Gene36671	Locus = hic_scaffold_5:39035187:39047158: −	5226	1742	189358.01	7.22	12	1
PeC3H82	PH02Gene36785	Locus = hic_scaffold_24:37308699:37311973: −	1005	335	38265.48	9.87	8	6
PeC3H83	PH02Gene36901	Locus = hic_scaffold_20:1950884:1958035: +	1137	379	41177.4	5.99	3	1
PeC3H84	PH02Gene36946	Locus = hic_scaffold_17:93181060:93192293: +	2067	689	75242.57	6.3	7	3
PeC3H85	PH02Gene39245	Locus = hic_scaffold_23:79674904:79682144: +	2646	882	95199.28	5.6	14	1
PeC3H86	PH02Gene39677	Locus = hic_scaffold_16:66766576:66770065: −	2100	700	75662.24	5.38	6	3
PeC3H87	PH02Gene40104	Locus = hic_scaffold_9:12635296:12636492: −	1011	337	36311.83	9.31	2	2
PeC3H88	PH02Gene40123	Locus = hic_scaffold_6:2254910:2269532: −	1401	467	49262.17	7.81	7	5
PeC3H89	PH02Gene40124	Locus = hic_scaffold_6:2209039:2229143: −	1374	458	48768.09	8.03	7	5
PeC3H90	PH02Gene40127	Locus = hic_scaffold_6:2120238:2131441: −	3063	1021	115144.84	6.3	14	2
PeC3H91	PH02Gene41303	Locus = hic_scaffold_24:2629434:2636718: −	1314	438	48611.57	5.28	5	1
PeC3H92	PH02Gene41304	Locus = hic_scaffold_24:2656565:2658166: −	876	292	32374.01	8.17	3	4
PeC3H93	PH02Gene41307	Locus = hic_scaffold_24:2189327:2203820: +	2073	691	76855.67	7.48	13	2
PeC3H94	PH02Gene41311	Locus = hic_scaffold_21:42803057:42804697: −	1641	547	58482.74	8.73	1	2
PeC3H95	PH02Gene42007	Locus = hic_scaffold_24:1257264:1258222: +	846	282	31391.01	7.08	2	3
PeC3H96	PH02Gene42009	Locus = hic_scaffold_4912:6226:13103: +	624	208	23091.42	6.99	4	2
PeC3H97	PH02Gene42261	Locus = hic_scaffold_6:69363645:69369366: −	1110	370	41618.05	8.13	8	4
PeC3H98	PH02Gene42371	Locus = hic_scaffold_14:80080938:80085791: −	1293	431	46788.84	8.76	7	5
PeC3H99	PH02Gene42383	Locus = hic_scaffold_8:61684248:61687121: −	909	303	31434.84	9.73	3	3
PeC3H100	PH02Gene42765	Locus = hic_scaffold_4:15352919:15354940: −	2022	674	71723.68	6.95	1	2
PeC3H101	PH02Gene43143	Locus = hic_scaffold_23:2926738:2930397: +	2169	723	81241.85	5.36	3	3
PeC3H102	PH02Gene43485	Locus = hic_scaffold_12:35408810:35410749: +	1788	596	62801.75	5.61	1	2
PeC3H103	PH02Gene43571	Locus = hic_scaffold_16:98850404:98854916: +	1383	461	49444.95	8.2	7	5
PeC3H104	PH02Gene43572	Locus = hic_scaffold_16:98806865:98817914: +	1422	474	49884.71	8.07	7	5
PeC3H105	PH02Gene44888	Locus = hic_scaffold_23:45392819:45397651: +	2820	940	100818.73	8.43	4	1
PeC3H106	PH02Gene44958	Locus = hic_scaffold_8:74157516:74163406: −	1137	379	43004.86	9.68	10	1
PeC3H107	PH02Gene45012	Locus = hic_scaffold_12:26410341:26443152: −	1329	443	47695.64	8.6	6	5
PeC3H108	PH02Gene45826	Locus = hic_scaffold_16:36751093:36752171: −	972	324	35569.23	8.85	2	2
PeC3H109	PH02Gene46111	Locus = hic_scaffold_12:22038066:22055363: +	1575	525	57211.13	6.91	11	5
PeC3H110	PH02Gene46793	Locus = hic_scaffold_22:50984696:50991093: +	1083	361	38258.18	6.82	2	3
PeC3H111	PH02Gene47633	Locus = hic_scaffold_3:6987231:6997525: −	5727	1909	205570.71	4.6	12	1
PeC3H112	PH02Gene47743	Locus = hic_scaffold_14:31493016:31493864: +	849	283	32720.39	9.48	1	2
PeC3H113	PH02Gene47893	Locus = hic_scaffold_7:1439853:1442862: −	1863	621	66287.64	6.66	7	1
PeC3H114	PH02Gene48093	Locus = hic_scaffold_24:2355849:2367348: +	996	332	37043.76	8.97	3	4
PeC3H115	PH02Gene48095	Locus = hic_scaffold_24:2291460:2312121: +	1353	451	51553.08	8.56	7	2
PeC3H116	PH02Gene48231	Locus = hic_scaffold_13:41293124:41298776: +	4137	1379	150077.01	4.49	1	6
PeC3H117	PH02Gene48688	Locus = hic_scaffold_21:47523541:47525855: −	1719	573	63436.28	6.01	7	1
PeC3H118	PH02Gene49477	Locus = hic_scaffold_24:2064661:2073723: −	567	189	21446.69	5.98	2	1
PeC3H119	PH02Gene49957	Locus = hic_scaffold_21:71838270:71841096: −	2058	686	72614.53	5.93	8	1

**FIGURE 1:**
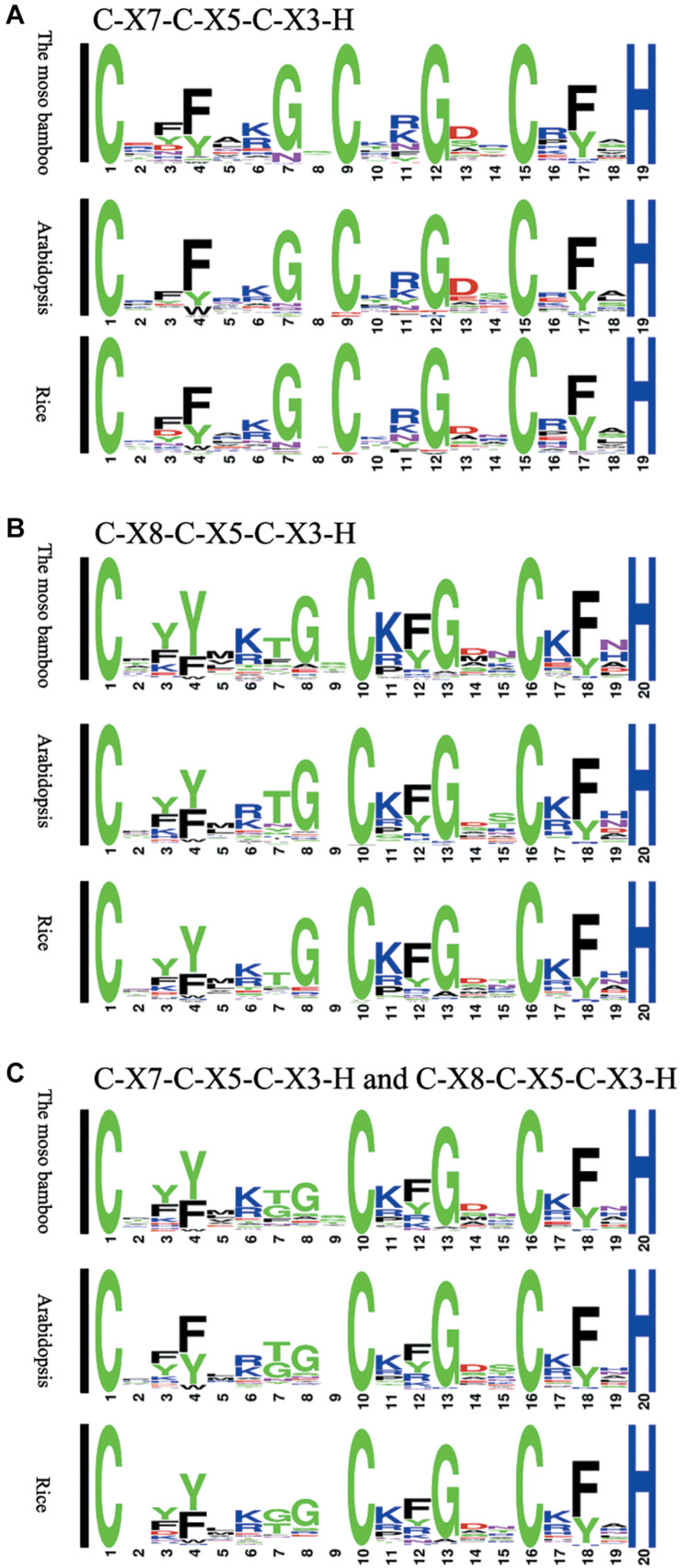
Sequence logos for common CCCH zinc finger motifs. **(A)** C-X_7_-C-X_5_-C-X_3_-H motifs of moso bamboo, rice and *Arabidopsis thaliana*. **(B)** C-X_8_-C-X_5_-C-X_3_-H motifs of moso bamboo, rice and *A. thaliana*. **(C)** C-X_7_-C-X_5_-C-X_3_-H and C-X_8_-C-X_5_-C-X_3_-H motifs of moso bamboo, rice and *A. thaliana*. The conservativeness of amino acids in different positions is indicated by the size of the characters.

We compared the sequences of 150 C-X_8_-C-X_5_-C-X_3_-H and 62 C-X_7_-C-X_5_-C-X_3_-H motifs separately, and then generated the sequence logos (Figure 1). To study the characteristics of the CCCH motifs of the *PeC3H* genes, the sequences were compared with CCCH motifs of rice and *A. thaliana*. In addition, the compound sequence logos of the two motifs were generated in the same way, and the motif sequences were highly conserved. Four of the amino acids in the three patterns were completely conserved, similar to the sequence diagrams provided by Pfam and SMART. There were also some differences in the sequence logos among the three plants. For example, lysine occurred more often at C1 + 6 than arginine in both moso bamboo and rice B motif logos, while the reverse case was observed in the *A. thaliana* B motif logo.

### Phylogenetic Relationships and Gene Architecture

To evaluate the phylogenetic relationships among the 119 predicted moso bamboo CCCH zinc finger proteins, we used the 119 putative protein sequences to construct an unrooted phylogenetic tree with 1,000 bootstrap replicates (Wang D. et al., 2008) (Supplementary Figure 1). We divided them into 13 subfamilies (CCCH A to M) based on bootstrap values above 100. However, the *Pe3H24* gene was not included in any of the 13 subfamilies because its bootstrap values with other genes were less than 100, which is a phenomenon that also occurs among the *CCCH* genes of other plants (Chai et al., 2012). Among the 13 subfamilies, subfamily L contained the greatest number of CCCH (29), followed by subfamilies C (25), F (15), A (12), and I (9). Both G and K subfamilies had six members, H and J had four members, and the B, D, E, and M had only two members each.

The MEME server identified 15 conserved motifs in the CCCH protein of moso bamboo (Supplementary Figure 2), and details of conserved amino acids are provided in Supplementary Table 1. Motif 1, 4, and 15 are typical CCCH motifs, and each *PeC3H* gene contains at least one CCCH motif (motif 1, 4, and 15). *PeC3H11*, *−15*, *−28*, *−33*, *−51*, and *−81* in I subfamily all contain SWIB motif (motif 13), and the SWI/SNF protein is required for proper protein complex formation in yeast. However, there are also some genetic differences within the same subfamily. In subfamily A, only *PeC3H110* contained motif 6, meanwhile, motif 5 only appears in *PeC3H4* and -*8*.

By analyzing the Gene Structure Display Server diagram (Supplementary Figure 3), we found that there were differences in the numbers of introns in the different genes, ranging from 0 to 13, with an average of 4. In total, 15 *PeC3H* genes had no introns, while 37 genes contained only one or two introns. Some subfamilies had similar numbers of introns in each member, but some subfamilies had significant differences. For example, *PeC3H9*, *−28*, and *−111* contain 1, 7 and 11 introns, respectively, and all they are all members of the I subfamily.

### Synteny Analysis of Moso Bamboo *CCCH* Genes

**FIGURE 2:**
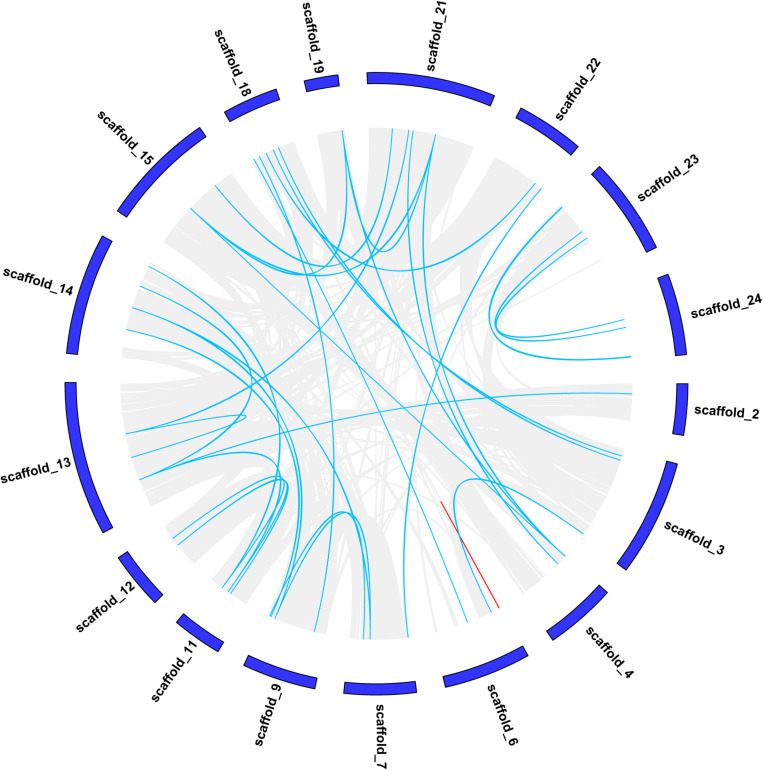
Chromosomal localizations and duplications of CCCH genes on moso bamboo chromosomes. The duplicated CCCH genes are indicated with blue lines and tandemly duplicated genes are marked with red.

The strongly conserved microsynteny of the *CCCH* gene was observed by comparing *CCCH* genes with other genes in moso bamboo (Figure 2), the synteny pairs details were shown in Supplementary Table 2. Pe_Scaffold1 did not contain any genes and is, therefore, not shown in Figure 2. In total, 63 collinear gene pairs were identified. We found that of the 119 *CCCH* genes, a pair of genes (*PeC3H88/-89*) in scaffold 6 were at a distance of less than 100 kb, which may be caused by a tandem duplication (Indicated by the red line) (Figure 2) (Lin et al., 2014). Both *PeC3H88* and *PeC3H89* belonged to the C subfamily, and their bootstrap values reach 1,000, indicating a highly conserved. The analysis of the collinear gene pairs of *CCCH* genes showed that 62 gene pairs remained in the conserved positions in the segmental duplicated blocks, indicating that gene duplication played an important role in the *CCCH* gene expansion of moso bamboo.

The gene duplication mechanisms of CCCH gene family in moso bamboo was studied by constructing a comparative syntenic maps with four representative species [one dicots (*Arabidopsis*) (Supplementary Figure 4A) and three monocots (*Brachypodium distachyon*, rice and maize) (Supplementary Figure 4B)]. The syntenic relationship between CCCH genes of bamboo and four species, the most is maize (118), followed by rice (107), *B. distachyon* (93) and *Arabidopsis* (2), indicating that in comparison with monocotyledonous plants, CCCH genes of moso bamboo show a high evolution divergence with dicotyledonous plants. *PeC3H4* and *PeC3H8* have syntenic pairs in all four plants, and these two genes may play a key role in the evolution of CCCH genes family. Through to *K*_a_/*K*_s_ ratios calculations of the CCCH genes synteny pairs (Supplementary Tables 2–6), *K*_a_/*K*_s_ < 1 of most of the synteny pairs, show that the moso bamboo CCCH genes family during evolution may experience a strong purifying selection pressure, and the *PeC3H* genes experienced a large-scale duplication event, probably 5.13–271.79 million years ago (MYA) by the formula *T* = *K*_s_/2λ (λ = 6.5 × 10^–9^) (Gaut et al., 1996).

### *Cis*-element Analysis

**FIGURE 3:**
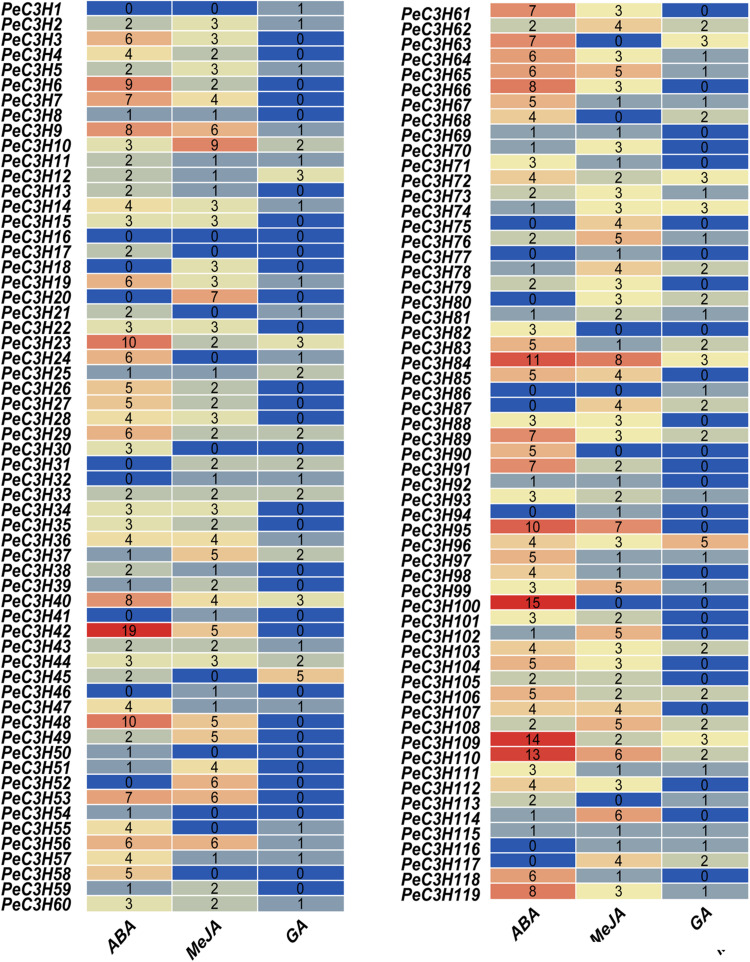
The position of moso bamboo CCCH genes abscisic acid (ABA), Me-JA, and GA in promoter. ABA was shown in red. Me-JA was shown in yellow. GA was shown in blue.

The promoter region of a gene usually contains multiple *cis*-elements that play key roles in responses to different stresses (Dung Tien et al., 2012). *Cis*-elements directly influence gene regulation involved in stress-responsive gene expression (Bilas et al., 2016). Various interactions between *cis*-acting elements and transcription factors function as molecular switches for transcription to determine transcription initiation events (Bilas et al., 2016). Therefore, identifying *cis*-acting elements in promoter region is very important to understand the role of transcription factors in stress response. The *cis*-acting elements in promoter regions of 119 *PeC3H* genes were detected to prepare study their regulatory mechanism. We focused on three types of *cis*-elements (ABA, Me-JA, and GA) (Figure 3 and Supplementary Table 7), and there were a large number of *cis*-elements related to these three hormones among the *PeC3H*s. ABA-responsive elements (ABREs), the *cis*-acting elements of ABA, existed in many *PeC3H* genes. Therefore, we speculated that most *PeC3H* genes were regulated by ABA stress responses. In total, 85.7% (102/119) of *PeC3H* gene promoter regions contained an ABA-responsive element (Figure 3 and Supplementary Table 7). Meanwhile, we found 440 ABRE elements in *PeC3Hs*, and the largest number of three elements. The CGTCA/TGACG-elements are *cis*-acting elements of the Me-JA response and have regulatory effects on plant leaf senescence (He et al., 2002). The CGTCA/TGACG-elements were found in promoter regions of 101 (84.8%) *PeC3Hs* (Supplementary Table 7), and 300 CGTCA/TGACG-elements in *PeC3Hs* were discovered. The GARE/P-box/TATC-box-element associated with GA was present in 62 of 119 (52.1%) promoter regions of *PeC3H*s, and 104 GARE/P-box/TATC-box-element in *PeC3Hs* were discovered. In addition, none of the three *cis*-acting elements was found in *PeC3H1*. The analysis of *cis*-acting elements in the *PeC3H*s will aid in further studies of the tolerance of moso bamboo.

### Expression Analysis of *PeC3H* Genes by qRT-PCR

**FIGURE 4:**
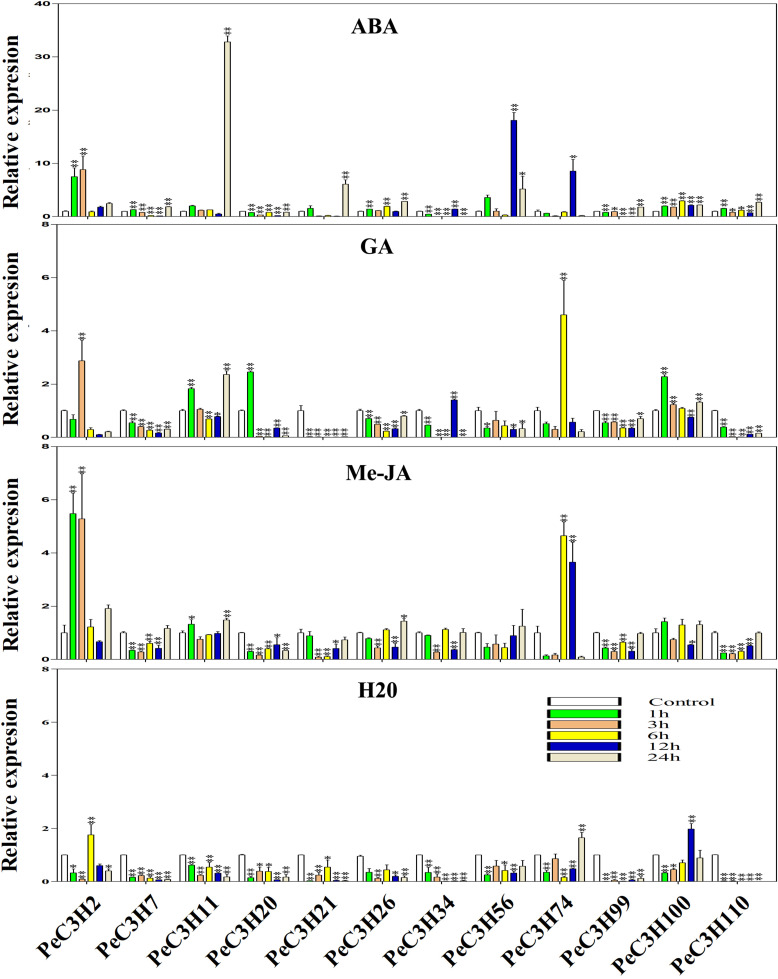
Expression levels of *PeC3Hs* under ABA, Me-JA, GA and H_2_O treatment by qRT-PCR. The *Y*-axis and *X*-axis indicate the relative expression levels and the time courses of plant hormone treatments, respectively. The experiment was performed using three biological and technical replicates each, and asterisks indicate significant difference compared to the transcription level of control groups, as determined by Student’s *t*-test (^∗^*p* < 0.05, ^∗∗^*p* < 0.01). Bars indicate standard error of the mean (SE).

There are some *CCCH* genes were positively or negatively regulated by ABA, GA and Me-JA in rice (Huang et al., 2012). Twelve genes (*PeC3H2*, *−7*,*−11*, *−20*, *−21*, *−26*, *−56*, *−34*, *−74*, *−99*, *−100*, and *−110*) were selected to study the expression level using qRT-PCR. The specific primers used in qRT-PCR analysis of these genes are shown in Supplementary Table 8. At the same time, the expression levels of these genes under water treatment were analyzed (Figure 4). Under water treatment, although the gene expression level changes, it is basically low expression.

The ABA treatment of moso bamboo, 11 genes reached their maximum expression levels at different times, except *PeC3H20* was inhibited compared with the control (Figure 4). In particular, gene *PeC3H11* had an expression level that was 32-fold greater than that of the control at 24 h (Figure 4).

After the GA treatment, the expression profiles of five genes (*PeC3H7*, *−21*, *−26*, *−56*, and *−99*) were suppressed, while those of other *PeC3H* genes were up-regulated, but the expression levels were lower than those of the control at some time. For example, *PeC3H74* reached its peak at 6 h and was greater than the control; however, its expression levels at other times were lower than the control (Figure 4). After the GA treatment, the expression levels of *PeC3H* genes were not significantly different from those of the control. For example, the expression levels of *PeC3H2* and *PeC3H56* were initially up-regulated, reaching their maximum levels, which were no more than fivefold those of the control. Furthermore, the expression levels of five genes (*PeC3H7*, *−11*, *−20*, *−100*, and *−110*) were greatest at 1 h.

Next, the expression levels of *CCCH* genes after Me-JA treatment was analyzed, and 4 genes (*PeC3H20*, *−21*, *−99*, and *−110*) were suppressed, while those of other *PeC3H* genes were up-regulated (Figure 4). At 24 h, the expression levels of four genes (*PeC3H7*, *−11*, *−26*, and *−56*) were the greatest. In addition, *PeC3H2* and *PeC3H100* reached the highest expression levels at 1 h. *PeC3H34* and *PeC3H74* reached their highest expression levels at 6 h.

Under ABA treatment, only the expression of *PeC3H20* was suppressed, but under the other two hormone treatments, at least 4 genes were suppressed. Only *PeC3H74* gene under the treatment of three hormones, the highest expression level exceeds fivefold, suggesting that this gene plays a role in resisting stress during plant growth and development. Thus, most *PeC3H* genes were up-regulated under stress treatments, indicating that they play key roles in abiotic and biotic stress responses.

### Subcellular Localization of *PeC3H74* and Transcriptional Activity

**FIGURE 5:**
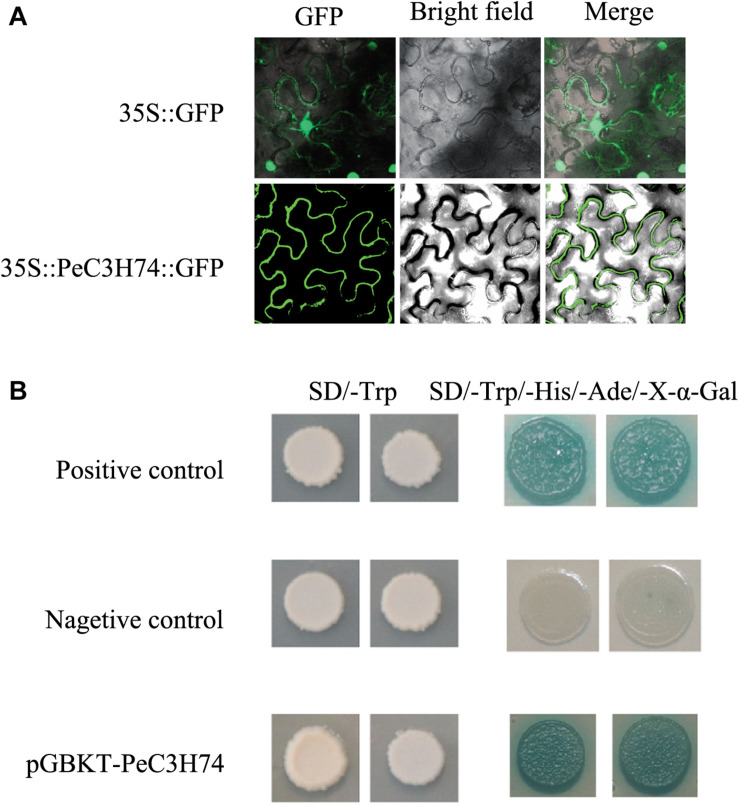
Subcellular localization of *PeC3H74* and transactivational analyses of PeC3H proteins in yeast Y2HGold strain. **(A)** The PeC3H74-GFP fusion proteins and GFP as a control were transiently expressed in *N. tabacum* leaves and observed under a fluorescence microscope. **(B)** The positive constructs, negative constructs, and fusion constructs were transformed into yeast Y2HGold strain and successively incubated in SD/–Trp media and SD–His/–Ade/–Trp plate supplemented with *X*-α-GAL.

To study the subcellular localization of *PeC3Hs*, a PeC3H-GFP vector was constructed and transiently expressed in *N. tabacum* leaves. 35S:GFP served as a control (Figure 5A). *PeC3H74* gene expression had higher induction under ABA, GA, and Me-JA treatment, so it was further analyzed. Based on the GFP signal, *PeC3H74* was localized to the cytomembrane.

The Y2H yeast strain was used to study the transcriptional activities of *PeC3H*s. The pGBKT7-PeC3H74, positive control plasmids pGBKT7-53 and pGADT7-T, and pGBKT7 (the negative control plasmid) transformed into the Y2H yeast strain, independently. The transformants were cultured on SD/–Trp medium, and they all produced white colonies (Figure 5B). On the SD–Ade/–His/–Trp/X-α-GAL medium, strains containing *PeC3H74* and positive control turned blue, while the negative control did not grow (Figure 5B). These results suggest that *PeC3H74* can function as a transcriptional activator.

### Overexpression of *PeC3H74* Enhanced Drought Tolerance in *Arabidopsis*

**FIGURE 6:**
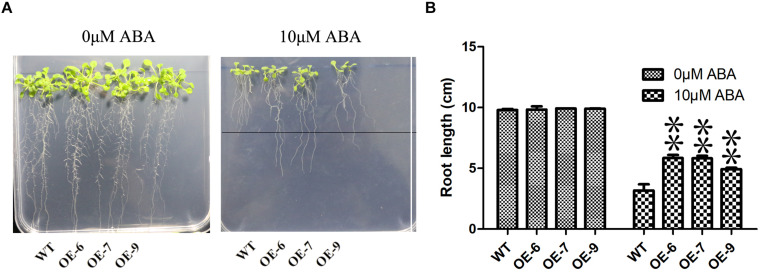
The *PeC3H74* gene improves resistance to ABA in *Arabidopsis thaliana*. **(A)** Root growth of WT and transgenic plants exposed to ABA. The seedlings were grown vertically in 1/2 MS for 4 days and then transferred to the 1/2 MS containing different concentrations of ABA. After 7 days, the representative images were taken and the root length in each line was measured. **(B)** Measurements of root lengths in ABA condition. The experiment was performed using three biological and technical replicates each, and asterisks indicate significant difference compared to the transcription level of control groups, as determined by Student’ s *t*-test (^∗^*p* < 0.05, ^∗∗^*p* < 0.01). Bars indicate standard error of the mean (SE).

Because the transgenic technology of moso bamboo is still immature, we transferred the *PeC3H74* gene into *A. thaliana* and studied whether *PeC3H74-OE* was related to drought stress through transgenic *Arabidopsis* strains (OE-6, OE-7, and OE-9). Leaves of transgenic *Arabidopsis* strains grown for 2 weeks drive GUS activity (Supplementary Figure 5). After 6 days of cultivation on 1/2 MS medium, *Arabidopsis* seedlings were placed on 1/2 MS medium containing different concentrations of ABA (0 and 10 μM). The root lengths of wild type and *PeC3H74-OE* on 0 μM ABA were not significantly different, while the root length of *PeC3H74-OE* on 10 μM ABA was significantly different from that of wild type (Figures 6A,B).

**FIGURE 7:**
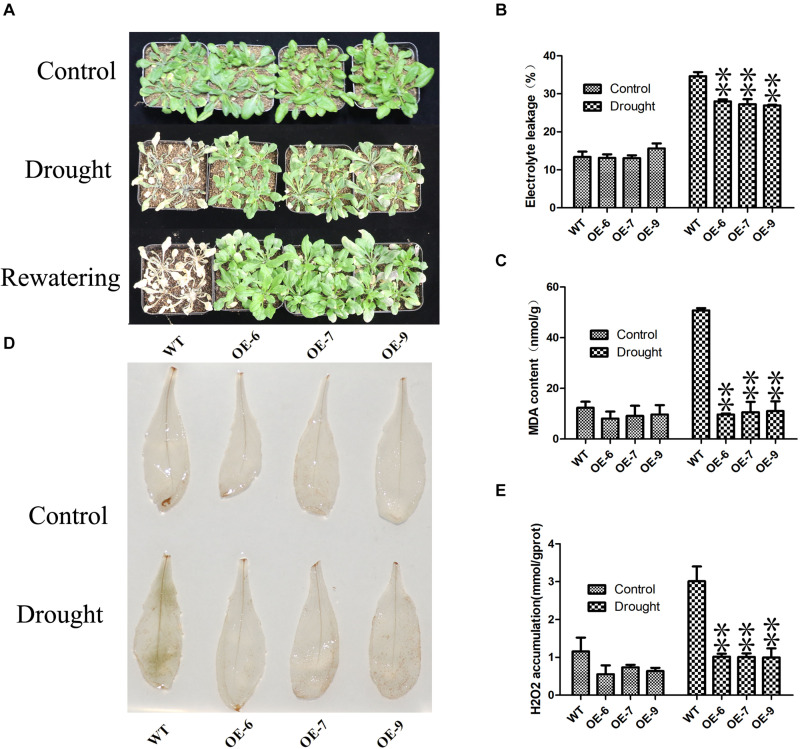
The *PeC3H74* gene improves drought resistance in *A. thaliana*. 3 weeks WT and transgenic plants were withheld water for 7 days to induce dehydration. After dehydration for 7 days, the representative images were taken **(A)**, Electrolyte leakage **(B)**, MDA content **(C)**, and H_2_O_2_ accumulation **(D,E)** were determined. The experiment was performed using three biological and technical replicates each, and asterisks indicate significant difference compared to the transcription level of control groups, as determined by Student’ s *t*-test (^∗^*p* < 0.05, ^∗∗^*p* < 0.01). Bars indicate standard error of the mean (SE).

Subsequently, we examined the drought tolerance of *PeC3H74-OE* strain. 3 weeks of WT and *PeC3H74-OE* plants were not irrigated for 7 days, WT withered more than *PeC3H74-OE* plants (Figure 7A). After re-watering for 3 days, all *PeC3H74-OE* plants were survived, the survival rate of the WT was only 16.7 percent. In addition, before drought treatment, the electrolyte leakage (EL) and malondialdehyde (MDA) of WT and *PeC3H74-OE* plants are not much different (Figures 7B,C). After 7 days of drought, the content of EL and MDA of WT Increased, significantly different from *PeC3H74-OE* plants. The results showed that after drought treatment, *PeC3H74-OE* plants suffered less membrane damage than WT. Drought stress can lead to accumulation of reactive oxygen species (ROS). Before drought treatment, DAB staining showed that H202 accumulated less in WT and *PeC3H74-OE* plants (Figure 7D). After 10 days of drought treatment, the accumulation of H_2_O_2_ in *PeC3H74-OE* plants was significantly less than that of WT. The detection of H_2_O_2_ content before and after treatment showed that after treatment, the difference in H_2_O_2_ between WT and *PeC3H74-OE* plants was significant, which was consistent with DAB staining results (Figure 7E). These results indicate that *PeC3H74* enhances the drought tolerance of *Arabidopsis*.

### ABA Sensitivity Analysis

**FIGURE 8:**
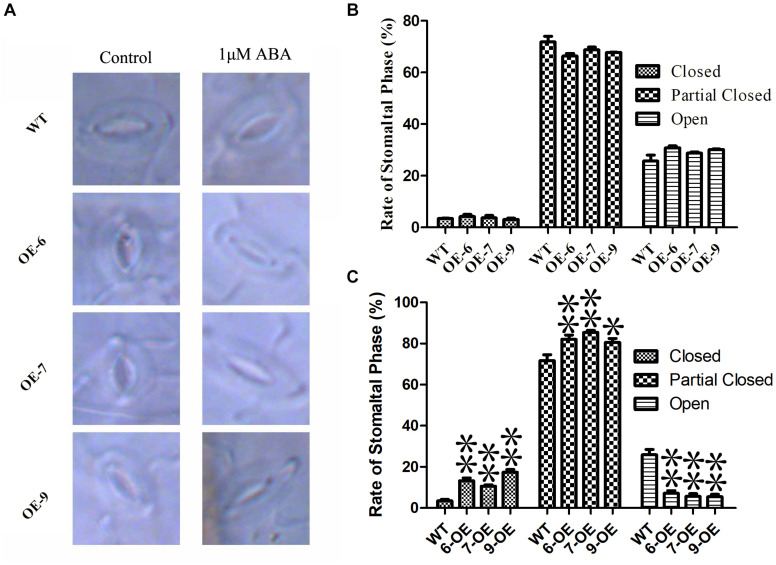
Abscisic acid sensitive and stomatal regulation of *PeC3H74* overexpression in *Arabidopsis*. **(A)** Typical phenotype of stomatal opening phase in transgenic lines and WT with or without ABA treatment. Percentages of the three types of stomata in transgenic lines and WT plants are calculated in stomatal-induced liquid with **(B)** or without **(C)** ABA treatment. The experiment was performed using three biological and technical replicates each, and asterisks indicate significant difference compared to the transcription level of control groups, as determined by Student’ s *t*-test (^∗^*p* < 0.05, ^∗∗^*p* < 0.01). Bars indicate standard error of the mean (SE).

The opening and closing of stomatal are affected by ABA, and the loss of water on the leaf surface is closely related to the regulation of stomatal (Osakabe et al., 2014). In order to determine the regulating effect of *PeC3H74-OE* on plant stomatal size. Observe the changes in stomatal size of WT and overexpressing strains without treatment or 1 μM ABA treatment, respectively. Observation by fluorescence microscopy revealed that the stomatal size of WT and overexpressing strains were not significantly different under untreated conditions. After 6 h of 1 μM ABA treatment, the rate of stomatal closure and partial closure of *PeC3H74-OE* plants was significantly higher than that of WT (Figures 8A–C). The results showed that *PeC3H74* may induce stomatal closure through ABA to achieve the purpose of drought resistance.

## Discussion

CCCH zinc finger proteins have been systematically analyzed in the model plants *A. thaliana* and rice (Wang D. et al., 2008). However, moso bamboo had not been studied. Here, 119 CCCH zinc finger protein were identified, which was a greater number than in *A. thaliana* and rice, and divided them into 13 subfamilies (A–M). Bioinformatics analyses found that the CCCH of moso bamboo had some characteristics that were similar to those of other species and some novel characteristics.

The sequence of the CCCH motif was highly conserved in moso bamboo. However, the *PeC3H* genes had different numbers of CCCH motifs, and the adjacent sequences and cysteine and histidine sequences of the motif members were also different. Previously, the CCCH family was defined as a group of zinc finger proteins having a C-X_6__–__14_-C-X_4__–__5_-C-X_3_-H motif, and CCCH proteins contain 1–6 copies of the CCCH zinc finger motif (Berg and Shi, 1996). However, there were different CCCH gene sequences in some plants. In *A. thaliana* and rice, the CCCH motifs were C-X_4__–__15_-C-X_4__–__6_-C-X_3_-H (Wang D. et al., 2008). In maize, *ZmC3H17*, which is a novel motif, was defined as C-X_17_-C-X_6_-C-X_3_-H, and we found a highly conserved CCCH zinc finger motif with 7 copies (Peng et al., 2012). In moso bamboo, *PeC3H13* and *PeC3H52* were defined as C-X_17_-C-X_6_-C-X_3_-H. In this study, moso bamboo, as in the previous result, has no more than six conserved CCCH motifs. In moso bamboo, 73.5% of all identified CCCH motifs are C-X_7__–__8_-C-X_5_-C-X_3_-H, and there were less of this particular motif than in *A. thaliana* (82.2%), rice (78.6%), maize (79.4%), and poplar (82.0%) CCCH genes families. In total, 287 CCCH zinc finger motifs were identified, which was more than in *A. thaliana* (152) and rice (150) (Wang D. et al., 2008), and the results were similar to the number of CCCH proteins.

Genes on corresponding scaffolds (syntenic genes) and corresponding sequences (collinear genes) were preserved to a certain extent in eukaryotic genomes during evolution. Synteny mainly refers to the similarity of arrangements in different genes. Microsynteny has been studied in both monocotyledonous and dicotyledonous plants (Deleu et al., 2007). There were 63 genes in moso bamboo that had no microsyntenic relationships with other genes, indicating that either they were ancient genes without detectable linkage to other CCCH genes or that they were formed through complete transposition and loss of their primogenitors (Wang Y. et al., 2015). Gene duplication is an important mechanism of biological evolution and plays an important role in allowing organism to cope with adverse environments (Bowers et al., 2003). Segmental duplication, tandem duplication and retro-positioning are the main mechanisms of gene duplication (Kong et al., 2007). Here, we found that most *CCCH* genes were distributed in duplicated blocks, indicating that segmental duplication was involved in *CCCH* gene amplification in moso bamboo.

Studying *cis*-elements in the upstream region of genes can be helpful (Lin et al., 2011a) to further understand and predict their transcriptional regulation (Ibraheem et al., 2010). ABA was produced in the vegetative tissue in the absence of water; therefore, under drought conditions, it promotes the expression of related genes (Yamaguchi-Shinozaki and Shinozaki, 2005). In rice, *OsC3H47* alters the drought resistance of rice by regulating ABA sensibilities (Wang W. et al., 2015). *AtTZF1* regulates ABA-mediated growth and stress responses by affecting gene expression (Lin et al., 2011b). ABREs play important roles in ABA-dependent gene expression (Yoshida et al., 2014). A promoter region analysis showed that several *PeC3H*s possess ABRE *cis*-elements (Figure 3 and Supplementary Table 7) (Yamauchi et al., 2007). For the 12 genes, except for the lower expression level of *PeC3H20* than the control, the maximum expression levels of other genes is higher than the control, so ABA sensitivity may be a common phenomenon of *PeC3H* genes.

GAs are tetracyclic diterpenes, which play roles in the growth and developmental stages of many plants, especially during the germination of seeds (Yamauchi et al., 2007). Ga1-3 and Ga2-1 are GA-deficient mutants with obvious seed germination defects (Yamauchi et al., 2007). In *A. thaliana*, the loss-of-function of *SOMNUS* (*AtTZF4*) results in elevated GA levels (Kim et al., 2008). *AtTZF1* regulates the GA-dependent growth of plants by affecting gene expression, which is a negative regulatory GA response (Lin et al., 2011a). Interestingly, the expression levels of five genes (*PeC3H7*, *−21*, *−26*, *−56*, and *−99*) were inhibited after GA treatments, and they may also have negative regulatory effects on GA levels (Figure 4). There may be both positive and negative regulation of *PeC3H* genes under GA treatment.

Me-JA is involved in plant immunity, and leaf senescence is regulated by JA (He et al., 2002). Transcriptome sequencing showed that the JA pathway is significantly active in age-dependent, dark-induced and starvation-induced leaf senescence (He et al., 2002). In rice, *OsDOS* can delay leaf senescence through the JA pathway (Kong et al., 2007). The expression of *GhTZF1* was significantly up-regulated after Me-JA treatments (Zhou et al., 2014). In the promoter analysis, we found that almost all *CCCH* genes contained a CGTCA or TGACG motif, which are Me-JA-responsive elements. qRT-PCR revealed that some *CCCH* genes in moso bamboo (*PeC3H2*, *−7*, *−11*, *−26*, *−34*, *−74*, and *−100*) had significantly increased expression levels at different times during the Me-JA treatment, indicating that they were positively regulated. *PeC3H* genes were positively regulated under Me-JA treatment, which may be a common phenomenon.

However, the expression levels of some genes treated with ABA, Me-JA, and GA were not as predicted (Figure 4). For example, the promoter regions of *PeC3H100* did not contain Me-JA-related *cis*-elements, but their expression levels increased during the Me-JA treatment. Thus, gene expression is a complex process that requires further study.

Some CCCH proteins localize in the nucleus, such as OsDOS, PEI1, AtSZF1, and SOMNUS, while some localize in the cytomembrane, such as HUA1, AtC3H49/AtTZF3, and AtC3H20/AtTZF2 (Lee et al., 2012). *PeC3H74* localized to the cytomembrane. In rice, OsLIC, a Novel CCCH-Type Zinc Finger Protein, displays transcriptional activation activity in yeast (Wang L. et al., 2008). Meanwhile, AtC3H17, in *A. thaliana*, showed transcriptional activation activity in yeast (Seok et al., 2018). In our research, the transactivation activity experiments with *PeC3H74* in a yeast system revealed that it was a transcriptional gene in yeast.

In plants, the CCCH gene plays an important (Ibraheem et al., 2010) role in all stages of growth and development, for example, seed germination (Kim et al., 2008), embryonic development (Li and Thomas, 1998) and secondary wall synthesis (Zhang et al., 2018), etc. However, there were few reports on the research of moso bamboo on abiotic stress. After we treated the transgenic *A. thaliana* for 3 weeks under drought conditions, *PeC3H74-OE* plants achieved a higher survival rate, as well as lower EL, MDA and H_2_O_2_ contents (Figures 7A–E). In addition, the roots of *PeCEH74-OE Arabidopsis* seedlings grew better on 10 μM ABA 1/2 MS medium (Figures 6A,B). ABA was a key factor in stomatal regulation. *OsC3H47*, an ABA-induced CCCH tandem zinc finger protein, regulates drought stresses by promoting ABA sensitivity in rice (Wang W. et al., 2015). Our research shows that under ABA treatment, transgenic *A. thaliana* contains more closure and partial closure stomatal than WT (Figures 8A–C). The above results show that the *PeC3H74* gene was quickly screened by bioinformatics. In addition, it may play a drought resistance function in plants through ABA-dependent pathways.

## Conclusion

In summary, the characteristics of *CCCH* gene families had been reported in some plants, such as *A. thaliana*, rice, tomato (*Solanum lycopersicum*) and poplar. However, no *CCCH* gene family studies had been reported in moso bamboo. Here, we identified 119 *CCCH* genes, and their phylogenetics, WebLogos, conserved motifs, divergence times, genetic structures and *cis*-acting components were analyzed. In addition, the subcellular localization and transcriptional activity of *PeC3H74* in moso bamboo were studied. The *PeC3H74* gene was quickly screened through bioinformatics. In addition, analysis of the phenotype and physiological and biochemical indicators of transgenic plants showed that *PeC3H74* gene may rely on the ABA pathway to play a positive role in regulating plant drought stress.

## Data Availability Statement

The datasets generated for this study can be found in online repositories. The names of the repository/repositories and accession number(s) can be found in the article/Supplementary Material.

## Author Contributions

FC carried out most of the experiments and bioinformatics analysis, and completed the main part of the manuscript. H-LL and KW guided some experiments. MW advised on the manuscript. Y-MG provided assistance with software usage. All authors contributed to the article and approved the submitted version.

## Conflict of Interest

The authors declare that the research was conducted in the absence of any commercial or financial relationships that could be construed as a potential conflict of interest.

## Footnotes

1 http://gigadb.org/

2 http://smart.embl-heidelberg.de/

3 https://pfam.sanger.ac.uk/

4 http://www.megasoftware.net/

5 http://meme.sdsc.edu/meme/itro.html

6 https://github.com/CJ-Chen/TBtools

## Supplementary Material

Supplementary Figure 1 Phylogenetic analysis of CCCH in moso bamboo.

Supplementary Figure 2 Schematic representation of the15 conserved motifs in *PeC3Hs*. Conserved motifs of the *PeC3Hs* were identified using the online MEME program based on 119 full-length amino acid sequences with the following parameters: maximum number of motifs, 10; maximum width, 100. The lengths and positions of different motifs in the protein sequences are identified by the lengths and positions of the different color blocks.

Supplementary Figure 3 Gene structures of CCCH in moso bamboo. Gene structures were performed using the Gene Structure Display Server online tool. Exons, introns, and untranslated regions (UTRs) are indicated by yellow rectangles, gray lines, and blue rectangles, respectively.

Supplementary Figure 4 Synteny analysis of CCCH genes between moso bamboo and **(A)** dicotyledonous plant *Arabidopsis thaliana*, **(B)** maize, rice and *Brachypodium distachyon*. Gray lines in the background indicate the collinear blocks within moso bamboo and other plant genomes, while the red lines highlight the syntenic CCCH gene pairs. Orange or green bars represent the chromosomes. The chromosome number is labeled at the top or bottom of each chromosome.

Supplementary Figure 5 Analysis of β-glucuronidase (GUS) activity driven of PeC3H74 in overexpression *Arabidopsis* leaves.

Supplementary Table 1 Detailed information on conserved amino acid sequences and motif lengths.

Supplementary Table 2 *K*_a_/*K*_s_ value for duplicate CCCH genes in moso bamboo.

Supplementary Table 3 *K*_a_/*K*_s_ value for duplicate CCCH genes between moso bamboo and *Arabidopsis*.

Supplementary Table 4 *K*_a_/*K*_s_ value for duplicate CCCH genes between moso bamboo and *Brachypodium distachyon*.

Supplementary Table 5 *K*_a_/*K*_s_ value for duplicate CCCH genes between moso bamboo and rice.

Supplementary Table 6 *K*_a_/*K*_s_ value for duplicate CCCH genes between moso bamboo and maize.

Supplementary Table 7 Kinds and numbers of known stress-related elements in the upstream regions of CCCH genes in moso bamboo.

Supplementary Table 8 The 12 gene primer sequences.
