# Supplementary figures and images for: Identification of CCCH Zinc Finger Proteins Family in Moso Bamboo (Phyllostachys edulis), and PeC3H74 Confers Drought Tolerance to Transgenic Plants
Source: Front Plant Sci. 2020 Nov 9;11:579255. doi: 10.3389/fpls.2020.579255 (PMC7680867; doi:10.3389/fpls.2020.579255)

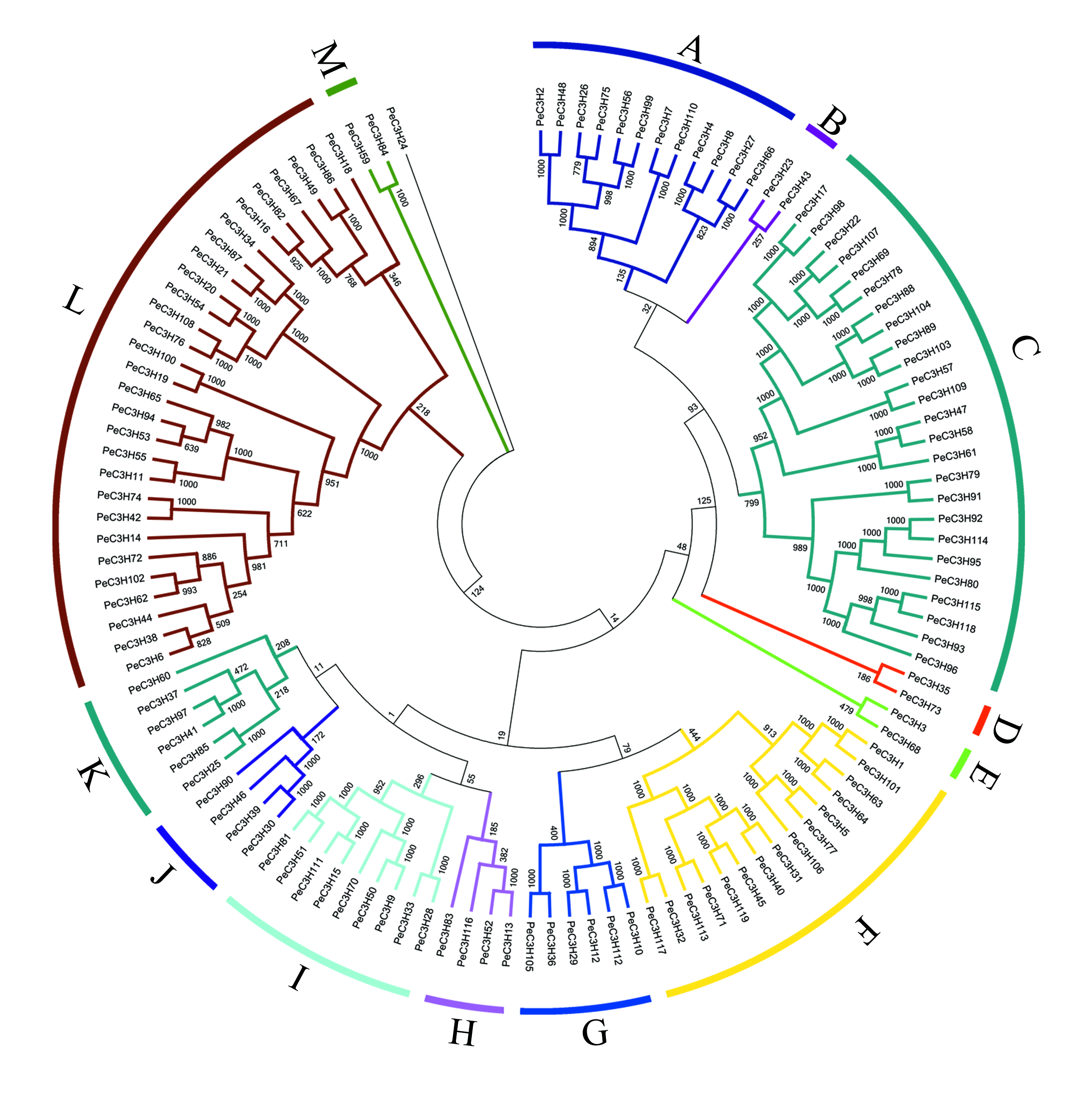

Supplement: Supplementary Figure 1 — Phylogenetic analysis of CCCH in moso bamboo. [file Image_1.TIF]

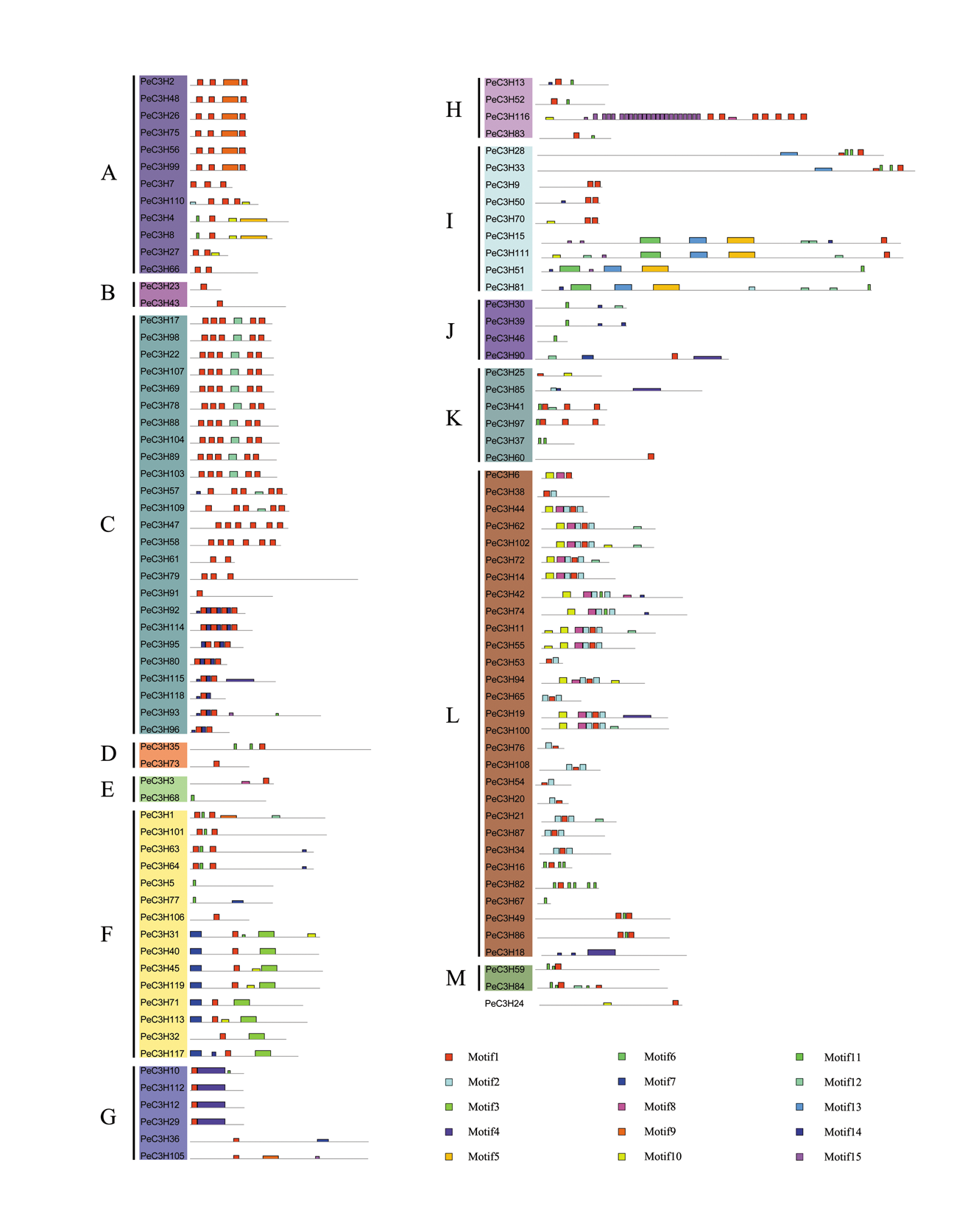

Supplement: Supplementary Figure 2 — Schematic representation of the15 conserved motifs in PeC3Hs. Conserved motifs of the PeC3Hs were identified using the online MEME program based on 119 full-length amino acid sequences with the following parameters: maximum number of motifs, 10; maximum width, 100. The lengths and positions of different motifs in the protein sequences are identified by the lengths and positions of the different color blocks. [file Image_2.TIF]

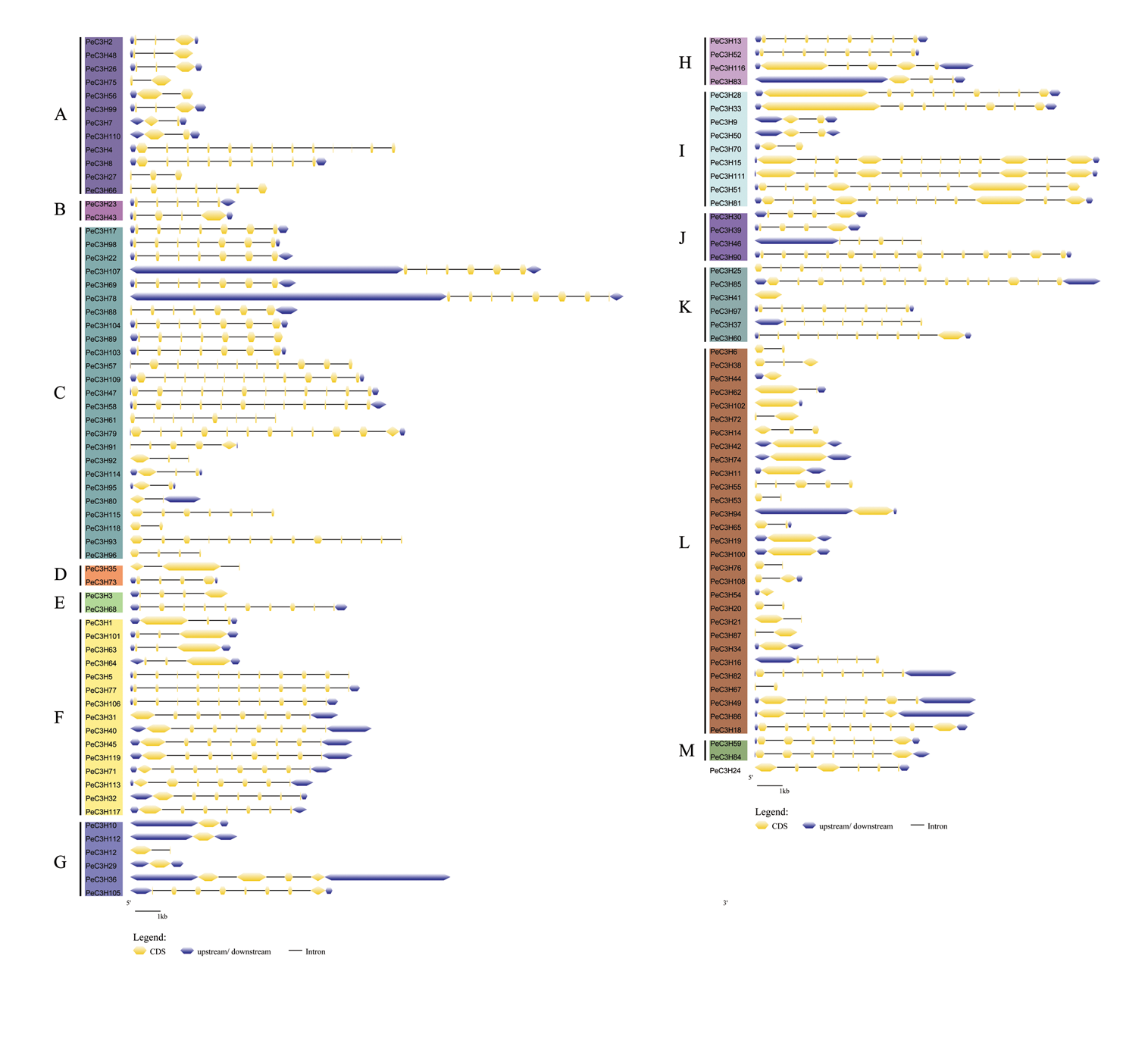

Supplement: Supplementary Figure 3 — Gene structures of CCCH in moso bamboo. Gene structures were performed using the Gene Structure Display Server online tool. Exons, introns, and untranslated regions (UTRs) are indicated by yellow rectangles, gray lines, and blue rectangles, respectively. [file Image_3.TIF]

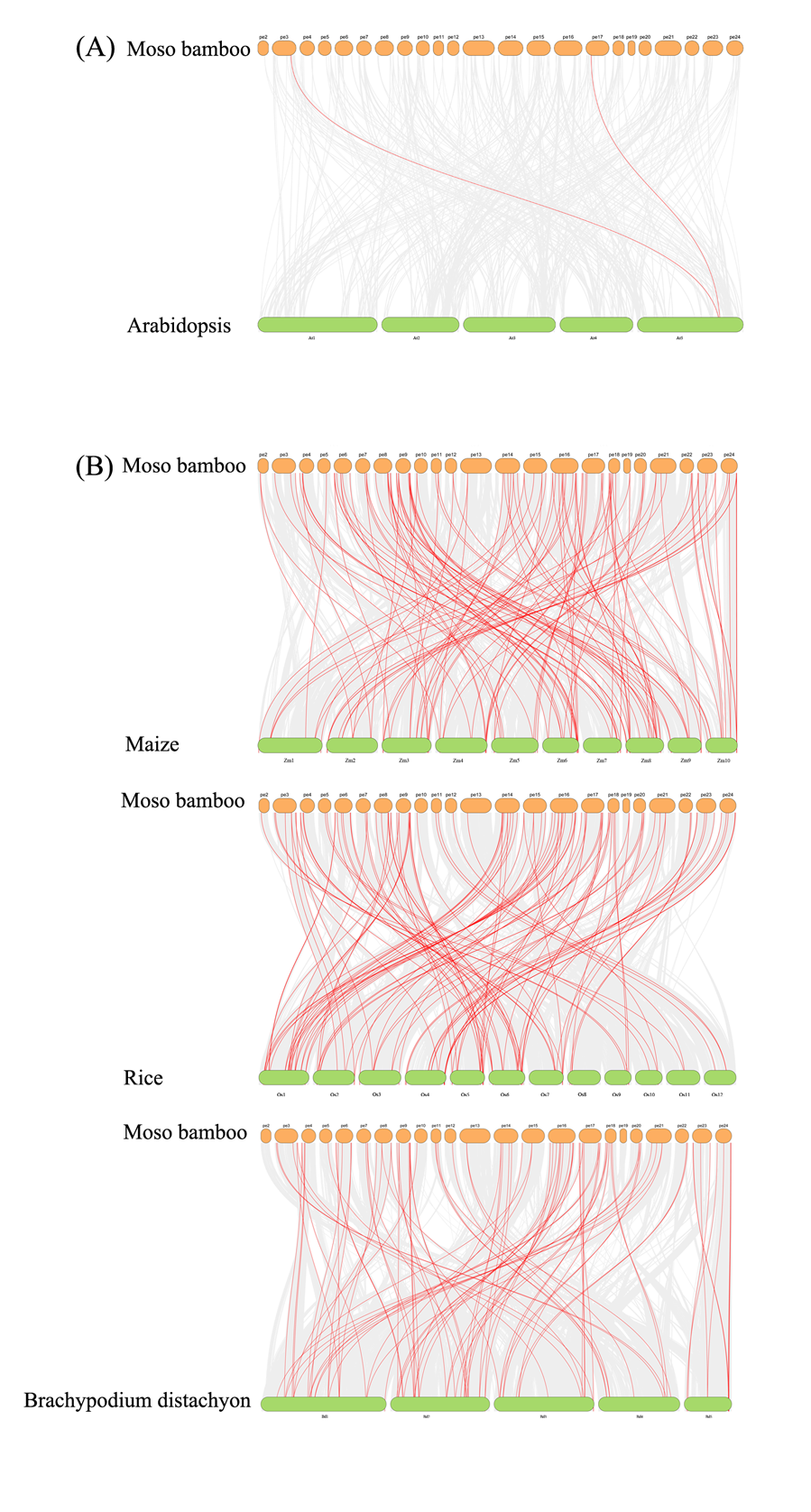

Supplement: Supplementary Figure 4 — Synteny analysis of CCCH genes between moso bamboo and (A) dicotyledonous plant Arabidopsis thaliana, (B) maize, rice and Brachypodium distachyon. Gray lines in the background indicate the collinear blocks within moso bamboo and other plant genomes, while the red lines highlight the syntenic CCCH gene pairs. Orange or green bars represent the chromosomes. The chromosome number is labeled at the top or bottom of each chromosome. [file Image_4.TIF]

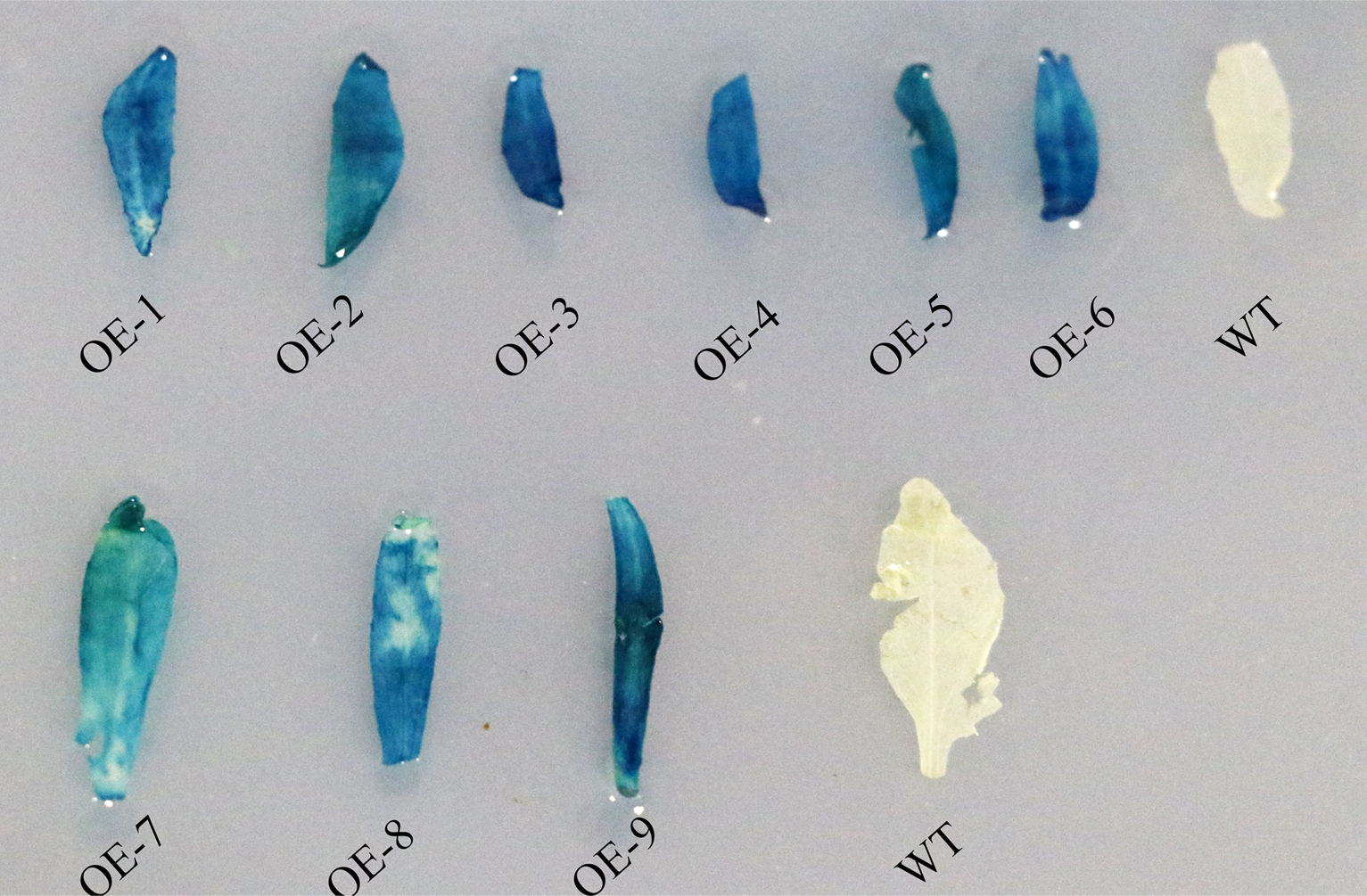

Supplement: Supplementary Figure 5 — Analysis of β-glucuronidase (GUS) activity driven of PeC3H74 in overexpression Arabidopsis leaves. [file Image_5.TIF]
