# Supplementary material for: Identification of CCCH Zinc Finger Proteins Family in Moso Bamboo (Phyllostachys edulis), and PeC3H74 Confers Drought Tolerance to Transgenic Plants
Source: Front Plant Sci. 2020 Nov 9;11:579255. doi: 10.3389/fpls.2020.579255 (PMC7680867; doi:10.3389/fpls.2020.579255)
Supplement: Supplementary Table 1 — Detailed information on conserved amino acid sequences and motif lengths. [file Table_1.DOC]

Table S1. Detailed information on conserved amino acid sequences and motif lengths

| **Motif** | **Length (aa)** | **Best possible match** |  |
| --- | --- | --- | --- |
| 1 | 49 | MYSFKVRPCSRAYSHDWTECPFVHPGENARRRDPRKYHYSCVPCPEFRK | ZnF_C3H1 |
| 2 | 11 | CKRGDSCKFAH |  |
| 3 | 88 | ANQRENVFPERPDQPECQYYMKTGDCKFGAVCKFHHPKERSIPKPNCVLSPLGLPLRPGEPPCTFYSRYGICKFGPNCKFDHPMGTLM | ZnF_C3H1/EGF_CA/FOLN/EGF_Lam/Low complexity region |
| 4 | 41 | GVFESWLHPAQYRTRLCKDGVACARRVCFFAHTPEELRVLN | ZnF_C3H1 |
| 5 | 80 | QIYLTFPADSTFREEDVSNYFSIYGPVHDVRIPYQQKRMFGFVTFVYPETVKLILAKGNPHFICDARVLVKPYKEKGKVP | RRM |
| 6 | 30 | YPERPGEPDCSYYVKTGSCKFGMNCKFNHP |  |
| 7 | 100 | MAEHLASIFGTEKDRVNCPFYFKIGACRHGDRCSRLHNRPSISPTJLLCNMYQRPDMITPGVDAQGNPIDPGKIQGDFEDFYEDIFDELSKYGEIETLNV | Low complexity region/DISIN/DISIN/ZnF_C3H1 |
| 8 | 15 | TGTCKFGDRCKFNHP |  |
| 9 | 100 | YKCGKKTTDIALEILNLDKKEIITIDTTSNQDFTEEECKRLRQSMKCGFIPRLKVGDVQEKAKIFQAVKVNDWJENEKQRLGHLRDRASDTGRRKELREC | Plus3/SH3 |
| 10 | 21 | NKPEYPLRPGEPDCSYYMKTG |  |
| 11 | 100 | DEEVCFICFDGGDLVVCDRRGCPKVYHPACIKRDDEFFKSKGKWNCGWHICSNCZKPVHHMCYTCTYSLCKVCIKZGKFFCVRGNKGFCDTCMGTILLIE | PHD/FANCL_C/RINGv/zf-AD/RING/KAZAL/PTI/FYVE/ZnF_RBZ/IB/EGF/ZnF_ZZ/BBOX |
| 12 | 41 | PPNSLPLKNHLLSLLGARKEYPPDPSLPDIKNGAYASDDFR | KH/PriCT_1 |
| 13 | 84 | FGASATAKISVDASLAGAIIGKGGVNTKQISRVTGAKLAIRDHESDPNLKNIELEGTFDQIKHASTMVKELIVSISGNAPPPAK | ANK/Low complexity region/Transmembrane region |
| 14 | 55 | DPNRASASDGATALHLAAAGGAPSAVAAVKLLLAAGADPTALDASGRRPADLIAL | PolyA |
| 15 | 57 | MDAYEATKVVFSRIQALDPDHAAKIMGLLLIQDHGEKEMIRLAFGPEALLQSVMAKA | PolyA |
| 16 | 21 | AAEKGEYPERPGZPDCSYYMK |  |
| 17 | 92 | DSKWASPELLDFVGHMRNGDKSVJSQFDVQALLLEYIKQNNLRDPRRKSQIICDYRLHSLFGKPRVGHFEMLKLLESHFLVSEVSPIDIDGN | SWIB |
| 18 | 29 | VSPRWPGPSSYAPMIVPQGLVPVPGWNPY |  |
| 19 | 100 | QSSGWAIPAQVANTSGQAQGAGNMNWGAALQGNASMGWGMMGQTNMNMSWGAPAQGAASYNMGLTMPTQPNAIPNMGWVAPNPGNTDMDMIWAATQGQGT |  |
| 20 | 100 | HMGHQTDSSNQRQQISTDMLAHSASKPAWRNDFQHDSVLEDGIDWSSNRTVQNQTSMKPAELPICSFAAAGNCPYGEECPHMHGDLCTTCGKMCLHPYRP | ZnF_C3H1/TAP_C/VWC/WAP/ZnF_C2HC |
