# Supplementary material for: Identification of CCCH Zinc Finger Proteins Family in Moso Bamboo (Phyllostachys edulis), and PeC3H74 Confers Drought Tolerance to Transgenic Plants
Source: Front Plant Sci. 2020 Nov 9;11:579255. doi: 10.3389/fpls.2020.579255 (PMC7680867; doi:10.3389/fpls.2020.579255)
Supplement: Supplementary Table 2 — Ka/Ks value for duplicate CCCH genes in moso bamboo. [file Table_2.DOC]

**Table S2. Ka/Ks value for duplicate CCCH genes in moso bamboo**

| Paralogous genes | Ka | Ks | Ka/Ks | Selection pressure |
| --- | --- | --- | --- | --- |
| PH02Gene02119&PH02Gene44958 | 0.270062075 | 0.714865757 | 0.377780125 | Purity selection |
| PH02Gene02576&PH02Gene46793 | 0.062206151 | 0.222606749 | 0.279444138 | Purity selection |
| PH02Gene03339&PH02Gene01488 | 0.042236418 | 0.11946102 | 0.353558159 | Purity selection |
| PH02Gene04361&PH02Gene25228 | 1.022773334 | 3.533242542 | 0.289471589 | Purity selection |
| PH02Gene04626&PH02Gene12613 | 0.062007127 | 0.265489132 | 0.233558061 | Purity selection |
| PH02Gene05151&PH02Gene47633 | 0.048624321 | 0.111641415 | 0.435540175 | Purity selection |
| PH02Gene05204&PH02Gene36785 | 0.08146983 | 0.129093614 | 0.631091093 | Purity selection |
| PH02Gene06968&PH02Gene42765 | 0.038365791 | 0.200495769 | 0.191354616 | Purity selection |
| PH02Gene08040&PH02Gene24845 | 0.029177099 | 0.066710615 | 0.437368164 | Purity selection |
| PH02Gene08203&PH02Gene40104 | 0.045275849 | 0.151729256 | 0.29839894 | Purity selection |
| PH02Gene08432&PH02Gene45012 | 0.024655533 | 0.104687881 | 0.235514682 | Purity selection |
| PH02Gene08432&PH02Gene30888 | 0.104171717 | 0.629489041 | 0.165486149 | Purity selection |
| PH02Gene10304&PH02Gene34123 | 0.115249514 | 0.197447096 | 0.5836982 | Purity selection |
| PH02Gene10304&PH02Gene42383 | 0.110050649 | 0.524563197 | 0.209794835 | Purity selection |
| PH02Gene10635&PH02Gene29104 | 0.434622686 | 0.672971072 | 0.645826698 | Purity selection |
| PH02Gene12814&PH02Gene49957 | 0.098758061 | 0.478343917 | 0.206458277 | Purity selection |
| PH02Gene12814&PH02Gene17992 | 0.026754073 | 0.175308579 | 0.152611314 | Purity selection |
| PH02Gene13668&PH02Gene11220 | 0.047048033 | 0.101888328 | 0.461760773 | Purity selection |
| PH02Gene14940&PH02Gene08203 | 0.128898726 | 0.444090275 | 0.290253432 | Purity selection |
| PH02Gene14940&PH02Gene40104 | 0.122219951 | 0.446243264 | 0.273886378 | Purity selection |
| PH02Gene16813&PH02Gene18149 | 0.697968352 | 1.601859098 | 0.435723937 | Purity selection |
| PH02Gene17257&PH02Gene12713 | 0.011033836 | 0.140854687 | 0.078334889 | Purity selection |
| PH02Gene19288&PH02Gene12814 | 0.097022101 | 0.454560201 | 0.213441697 | Purity selection |
| PH02Gene19288&PH02Gene49957 | 0.035675796 | 0.13168483 | 0.270918037 | Purity selection |
| PH02Gene19288&PH02Gene17992 | 0.098891041 | 0.45062461 | 0.219453264 | Purity selection |
| PH02Gene19939&PH02Gene17257 | 0.260312243 | 1.300351941 | 0.200185991 | Purity selection |
| PH02Gene19939&PH02Gene12713 | 0.287029364 | 1.192723929 | 0.240650294 | Purity selection |
| PH02Gene19983&PH02Gene26949 | 0.044234239 | 0.109741546 | 0.403076504 | Purity selection |
| PH02Gene20573&PH02Gene00385 | 0.030977855 | 0.162396234 | 0.19075476 | Purity selection |
| PH02Gene22177&PH02Gene39677 | 0.097069733 | 0.130489649 | 0.743888376 | Purity selection |
| PH02Gene22259&PH02Gene04182 | 0.051468625 | 0.09393925 | 0.547892654 | Purity selection |
| PH02Gene23823&PH02Gene04944 | 0.0233119 | 0.124488853 | 0.187260943 | Purity selection |
| PH02Gene27671&PH02Gene43485 | 0.02917689 | 0.231405053 | 0.126085793 | Purity selection |
| PH02Gene28052&PH02Gene27920 | 0.060516683 | 0.110915997 | 0.545608254 | Purity selection |
| PH02Gene30888&PH02Gene42371 | 0.292024061 | 1.266332628 | 0.230606125 | Purity selection |
| PH02Gene30888&PH02Gene34953 | 0.050329707 | 0.167941708 | 0.299685574 | Purity selection |
| PH02Gene32013&PH02Gene22259 | 0.205994882 | 0.355114057 | 0.580080901 | Purity selection |
| PH02Gene32013&PH02Gene04182 | 0.221420188 | 0.364605936 | 0.607286295 | Purity selection |
| PH02Gene32078&PH02Gene47893 | 0.038842362 | 0.108850115 | 0.356842633 | Purity selection |
| PH02Gene33725&PH02Gene18259 | 0.02075729 | 0.163674164 | 0.126820808 | Purity selection |
| PH02Gene34123&PH02Gene42383 | 0.204295508 | 0.72516107 | 0.281724319 | Purity selection |
| PH02Gene34597&PH02Gene45826 | 0.099587299 | 0.33564892 | 0.296700788 | Purity selection |
| PH02Gene34597&PH02Gene08040 | 0.353672823 | 0.594123863 | 0.595284661 | Purity selection |
| PH02Gene34597&PH02Gene24845 | 0.183715487 | 0.473767097 | 0.387775952 | Purity selection |
| PH02Gene34666&PH02Gene02119 | 0.03313792 | 0.111315004 | 0.297695 | Purity selection |
| PH02Gene34666&PH02Gene44958 | 0.272561797 | 0.671963286 | 0.405620072 | Purity selection |
| PH02Gene36946&PH02Gene27145 | 0.138078163 | 0.603139621 | 0.228932336 | Purity selection |
| PH02Gene42261&PH02Gene18149 | 1.327058688 | 2.275127282 | 0.583289866 | Purity selection |
| PH02Gene42371&PH02Gene05739 | 0.024193526 | 0.081446913 | 0.297046562 | Purity selection |
| PH02Gene43143&PH02Gene00351 | 0.048979057 | 0.149720413 | 0.327136801 | Purity selection |
| PH02Gene43572&PH02Gene40124 | 0.22704952 | 0.687987492 | 0.330019837 | Purity selection |
| PH02Gene44888&PH02Gene16079 | 0.050331318 | 0.113955208 | 0.441676325 | Purity selection |
| PH02Gene45826&PH02Gene08040 | 0.623205478 | 1.109274414 | 0.561813624 | Purity selection |
| PH02Gene45826&PH02Gene24845 | 0.215125692 | 0.576277951 | 0.373301965 | Purity selection |
| PH02Gene47743&PH02Gene04254 | 0.062032118 | 0.525395466 | 0.118067479 | Purity selection |
| PH02Gene48688&PH02Gene13318 | 0.610728435 | NaN | NaN |  |
| PH02Gene49957&PH02Gene17992 | 0.101742291 | 0.485797276 | 0.209433638 | Purity selection |
| PH02Gene17017&PH02Gene27671 | 0.542948796 | 0.661461658 | 0.820831849 | Purity selection |
| PH02Gene23886&PH02Gene28235 | 0.123501061 | 0.182331737 | 0.677342646 | Purity selection |
| PH02Gene25228&PH02Gene42383 | 0.034540638 | 0.144777803 | 0.238576892 | Purity selection |
| PH02Gene26317&PH02Gene45012 | 0.635186286 | 2.031481964 | 0.312671389 | Purity selection |
| PH02Gene28235&PH02Gene41311 | 0.185987711 | 0.349469033 | 0.532200835 | Purity selection |
| PH02Gene40123&PH02Gene40124 | 0.219436029 | 0.751083139 | 0.29215944 | Purity selection |
