# Supplementary material for: Identification of CCCH Zinc Finger Proteins Family in Moso Bamboo (Phyllostachys edulis), and PeC3H74 Confers Drought Tolerance to Transgenic Plants
Source: Front Plant Sci. 2020 Nov 9;11:579255. doi: 10.3389/fpls.2020.579255 (PMC7680867; doi:10.3389/fpls.2020.579255)
Supplement: Supplementary Table 3 — Ka/Ks value for duplicate CCCH genes between moso bamboo and Arabidopsis. [file Table_3.DOC]

**Table S3. Ka/Ks value for duplicate CCCH genes between moso bamboo and *Arabidopsis***

| PeC3H Gene ID | AtC3H Gene ID | Ka | Ks | Ka/Ks | Selection pressure |
| --- | --- | --- | --- | --- | --- |
| PH02Gene01488 | AT5G51980.1 | 0.30728601 | NaN | NaN |  |
| PH02Gene03339 | AT5G51980.1 | 0.295698947 | NaN | NaN |  |
