# Supplementary material for: Identification of CCCH Zinc Finger Proteins Family in Moso Bamboo (Phyllostachys edulis), and PeC3H74 Confers Drought Tolerance to Transgenic Plants
Source: Front Plant Sci. 2020 Nov 9;11:579255. doi: 10.3389/fpls.2020.579255 (PMC7680867; doi:10.3389/fpls.2020.579255)
Supplement: Supplementary Table 4 — Ka/Ks value for duplicate CCCH genes between moso bamboo and Brachypodium distachyon. [file Table_4.DOC]

**Table S4. Ka/Ks value for duplicate CCCH genes between moso bamboo and *Brachypodium distachyon***

| PeC3H Gene ID | BdC3H Gene ID | Ka | Ks | Ka/Ks | Selection pressure |
| --- | --- | --- | --- | --- | --- |
| PH02Gene06968 | Bradi1g24270 | 0.071178728 | 0.439655164 | 0.161896717 | Purifying selection |
| PH02Gene19288 | Bradi1g63570 | 0.113717615 | 0.266185134 | 0.427212496 | Purifying selection |
| PH02Gene22705 | Bradi1g77760 | 0.182458533 | 0.377134975 | 0.483801677 | Purifying selection |
| PH02Gene19288 | Bradi1g17510 | 0.147517868 | 0.478034723 | 0.308592369 | Purifying selection |
| PH02Gene34666 | Bradi1g32977 | 0.376851545 | 0.741431008 | 0.508275944 | Purifying selection |
| PH02Gene16813 | Bradi1g42720 | 0.776135355 | 1.259567055 | 0.616192168 | Purifying selection |
| PH02Gene10304 | Bradi1g35920 | 0.12182782 | 0.662550725 | 0.18387697 | Purifying selection |
| PH02Gene02119 | Bradi1g32977 | 0.403477259 | 0.668652109 | 0.603418808 | Purifying selection |
| PH02Gene34123 | Bradi1g35920 | 0.198788403 | 0.92058387 | 0.215937309 | Purifying selection |
| PH02Gene18259 | Bradi1g11760 | 0.058269625 | 0.381138583 | 0.152883039 | Purifying selection |
| PH02Gene49957 | Bradi1g63570 | 0.12369672 | 0.279119316 | 0.443167896 | Purifying selection |
| PH02Gene49957 | Bradi1g17510 | 0.128685699 | 0.518621457 | 0.248130302 | Purifying selection |
| PH02Gene48688 | Bradi1g23370 | 0.904762222 | NaN | NaN |  |
| PH02Gene19939 | Bradi1g48140 | 0.279663363 | 1.719507761 | 0.162641524 | Purifying selection |
| PH02Gene13318 | Bradi1g23370 | 0.443994067 | 0.891733832 | 0.497899767 | Purifying selection |
| PH02Gene42765 | Bradi1g24270 | 0.074670283 | 0.446257852 | 0.16732542 | Purifying selection |
| PH02Gene17992 | Bradi1g17510 | 0.076163117 | 0.401213877 | 0.189831712 | Purifying selection |
| PH02Gene17992 | Bradi1g63570 | 0.168878962 | 0.534146532 | 0.316165981 | Purifying selection |
| PH02Gene29764 | Bradi1g34507 | 0.099343093 | 0.294684232 | 0.337117096 | Purifying selection |
| PH02Gene42261 | Bradi1g42720 | 0.062290239 | 0.323577095 | 0.192505093 | Purifying selection |
| PH02Gene27145 | Bradi1g32560 | 0.059243981 | 0.474903964 | 0.124749392 | Purifying selection |
| PH02Gene17257 | Bradi1g48140 | 0.037985781 | 0.290952209 | 0.130556772 | Purifying selection |
| PH02Gene44958 | Bradi1g32977 | 0.238914235 | 0.41482296 | 0.575942651 | Purifying selection |
| PH02Gene18149 | Bradi1g42720 | 0.070030553 | 0.288885328 | 0.242416441 | Purifying selection |
| PH02Gene12713 | Bradi1g48140 | 0.044496566 | 0.349913195 | 0.127164585 | Purifying selection |
| PH02Gene23823 | Bradi2g54220 | 0.05341919 | 0.337269644 | 0.158387187 | Purifying selection |
| PH02Gene42371 | Bradi2g58770 | 0.055491106 | 0.259707178 | 0.21366797 | Purifying selection |
| PH02Gene19983 | Bradi2g43600 | 0.152702572 | 0.346967641 | 0.440106091 | Purifying selection |
| PH02Gene47743 | Bradi2g46080 | 0.039686549 | 0.456469849 | 0.086942323 | Purifying selection |
| PH02Gene47743 | Bradi2g16520 | 0.096829613 | 0.957677511 | 0.101108789 | Purifying selection |
| PH02Gene14940 | Bradi2g33270 | 0.239594121 | 0.56869352 | 0.421306227 | Purifying selection |
| PH02Gene04944 | Bradi2g54220 | 0.041279947 | 0.361549341 | 0.114175142 | Purifying selection |
| PH02Gene05739 | Bradi2g58770 | 0.048589179 | 0.25519771 | 0.190398177 | Purifying selection |
| PH02Gene29104 | Bradi2g04620 | 0.305571543 | 0.631873388 | 0.483596158 | Purifying selection |
| PH02Gene14940 | Bradi2g05750 | 0.117555528 | 0.401190621 | 0.293016641 | Purifying selection |
| PH02Gene43572 | Bradi2g09340 | 0.242365844 | 0.719498828 | 0.336853702 | Purifying selection |
| PH02Gene26949 | Bradi2g43600 | 0.149136586 | 0.356856499 | 0.417917528 | Purifying selection |
| PH02Gene32013 | Bradi2g45090 | 0.107706797 | 0.286991462 | 0.375296172 | Purifying selection |
| PH02Gene45826 | Bradi2g19270 | 0.336281797 | 0.818938277 | 0.410631432 | Purifying selection |
| PH02Gene39677 | Bradi2g41540 | 0.175800529 | 0.326583322 | 0.538302226 | Purifying selection |
| PH02Gene32013 | Bradi2g15670 | 0.256296709 | 0.393229943 | 0.651773125 | Purifying selection |
| PH02Gene04361 | Bradi2g38247 | 0.074809892 | 0.429677613 | 0.174107027 | Purifying selection |
| PH02Gene08203 | Bradi2g05750 | 0.200166279 | 0.608197662 | 0.329113858 | Purifying selection |
| PH02Gene08203 | Bradi2g33270 | 0.159841365 | 0.504942446 | 0.316553631 | Purifying selection |
| PH02Gene08811 | Bradi2g08905 | 0.104391316 | 0.269604248 | 0.387202046 | Purifying selection |
| PH02Gene40124 | Bradi2g09340 | 0.106542931 | 0.317772703 | 0.335280312 | Purifying selection |
| PH02Gene22259 | Bradi2g15670 | 0.172655311 | 0.237417838 | 0.727221308 | Purifying selection |
| PH02Gene08040 | Bradi2g19270 | 0.142401572 | 0.429752152 | 0.331357438 | Purifying selection |
| PH02Gene22259 | Bradi2g45090 | 0.270388111 | 0.375859295 | 0.719386521 | Purifying selection |
| PH02Gene25228 | Bradi2g38247 | 0.078535107 | 0.420088862 | 0.186948796 | Purifying selection |
| PH02Gene40104 | Bradi2g05750 | 0.187587259 | 0.590268522 | 0.317799869 | Purifying selection |
| PH02Gene04182 | Bradi2g15670 | 0.182768538 | 0.26435691 | 0.691370382 | Purifying selection |
| PH02Gene04254 | Bradi2g16520 | 0.043002919 | 0.448734808 | 0.095831475 | Purifying selection |
| PH02Gene24845 | Bradi2g19270 | 0.103826191 | 0.384873713 | 0.269766906 | Purifying selection |
| PH02Gene40104 | Bradi2g33270 | 0.105113491 | 0.450590499 | 0.233279422 | Purifying selection |
| PH02Gene04182 | Bradi2g45090 | 0.278882805 | 0.362115387 | 0.770149004 | Purifying selection |
| PH02Gene04254 | Bradi2g46080 | 0.079055955 | 0.73420579 | 0.107675471 | Purifying selection |
| PH02Gene34666 | Bradi3g04650 | 0.083113674 | 0.35364194 | 0.235022107 | Purifying selection |
| PH02Gene10304 | Bradi3g06940 | 0.066898829 | 0.251894196 | 0.26558305 | Purifying selection |
| PH02Gene03339 | Bradi3g51650 | 0.041285239 | 0.277394439 | 0.148832252 | Purifying selection |
| PH02Gene16813 | Bradi3g53502 | 0.709010485 | 2.289084455 | 0.309735398 | Purifying selection |
| PH02Gene18357 | Bradi3g53880 | 0.074836806 | 0.350198573 | 0.213698204 | Purifying selection |
| PH02Gene28052 | Bradi3g60617 | 0.147612279 | 0.389987859 | 0.378504806 | Purifying selection |
| PH02Gene02576 | Bradi3g16360 | 0.082789349 | 0.402777601 | 0.205546059 | Purifying selection |
| PH02Gene02119 | Bradi3g04650 | 0.084175994 | 0.349730076 | 0.240688461 | Purifying selection |
| PH02Gene34123 | Bradi3g06940 | 0.167871027 | 0.428623994 | 0.391651025 | Purifying selection |
| PH02Gene46793 | Bradi3g16360 | 0.054937237 | 0.375019787 | 0.14649157 | Purifying selection |
| PH02Gene15731 | Bradi3g14480 | 0.127666661 | 0.334649679 | 0.381493451 | Purifying selection |
| PH02Gene01488 | Bradi3g51650 | 0.066981425 | 0.282131755 | 0.23741186 | Purifying selection |
| PH02Gene27920 | Bradi3g60617 | 0.137753997 | 0.336240403 | 0.409689008 | Purifying selection |
| PH02Gene36671 | Bradi3g30620 | 0.129277912 | 0.361891386 | 0.357228485 | Purifying selection |
| PH02Gene44958 | Bradi3g04650 | 0.316218014 | 0.946920803 | 0.333943465 | Purifying selection |
| PH02Gene18149 | Bradi3g53502 | 0.226576284 | 0.726905518 | 0.311699771 | Purifying selection |
| PH02Gene27671 | Bradi4g06290 | 0.062293966 | 0.452518435 | 0.137660614 | Purifying selection |
| PH02Gene08432 | Bradi4g08050 | 0.043815691 | 0.246056782 | 0.178071461 | Purifying selection |
| PH02Gene26317 | Bradi4g39137 | 0.130054645 | 0.380564393 | 0.341741496 | Purifying selection |
| PH02Gene43485 | Bradi4g06290 | 0.066650051 | 0.458933397 | 0.145228156 | Purifying selection |
| PH02Gene30888 | Bradi4g19010 | 0.061551598 | 0.278909029 | 0.220687003 | Purifying selection |
| PH02Gene30888 | Bradi4g08050 | 0.109164942 | 0.645028487 | 0.169240498 | Purifying selection |
| PH02Gene04626 | Bradi4g33870 | 0.116140622 | 0.475176981 | 0.244415505 | Purifying selection |
| PH02Gene05151 | Bradi4g35977 | 0.129675439 | 0.327462324 | 0.396001095 | Purifying selection |
| PH02Gene12613 | Bradi4g33870 | 0.050555746 | 0.406769826 | 0.124285881 | Purifying selection |
| PH02Gene47633 | Bradi4g35977 | 0.132806552 | 0.342953072 | 0.387244096 | Purifying selection |
| PH02Gene05204 | Bradi5g10750 | 0.154159381 | 0.304638594 | 0.506040222 | Purifying selection |
| PH02Gene20573 | Bradi5g25340 | 0.080873147 | 0.444316251 | 0.182017081 | Purifying selection |
| PH02Gene43143 | Bradi5g25760 | 0.104764851 | 0.30726562 | 0.340958586 | Purifying selection |
| PH02Gene39245 | Bradi5g02300 | 0.090307585 | 0.299452521 | 0.30157564 | Purifying selection |
| PH02Gene00402 | Bradi5g25100 | 0.114399795 | 0.358936362 | 0.318718879 | Purifying selection |
| PH02Gene00385 | Bradi5g25340 | 0.082317232 | 0.42424967 | 0.194030161 | Purifying selection |
| PH02Gene00351 | Bradi5g25760 | 0.110110794 | 0.309459069 | 0.355816989 | Purifying selection |
| PH02Gene36785 | Bradi5g10750 | 0.108694246 | 0.338988843 | 0.320642548 | Purifying selection |
| PH02Gene09684 | Bradi5g01730 | 1.140815751 | NaN | NaN |  |
