# Supplementary material for: Identification of CCCH Zinc Finger Proteins Family in Moso Bamboo (Phyllostachys edulis), and PeC3H74 Confers Drought Tolerance to Transgenic Plants
Source: Front Plant Sci. 2020 Nov 9;11:579255. doi: 10.3389/fpls.2020.579255 (PMC7680867; doi:10.3389/fpls.2020.579255)
Supplement: Supplementary Table 5 — Ka/Ks value for duplicate CCCH genes between moso bamboo and rice. [file Table_5.DOC]

**Table S5. Ka/Ks value for duplicate CCCH genes between moso bamboo and rice**

| PeC3H Gene ID | OsC3H Gene ID | Ka | Ks | Ka/Ks | Selection pressure |
| --- | --- | --- | --- | --- | --- |
| PH02Gene22177 | LOC_Os01g39100 | 0.209314403 | 0.416345484 | 0.502742101 | Purifying selection |
| PH02Gene19983 | LOC_Os01g42970 | 0.146381914 | 0.314924063 | 0.464816541 | Purifying selection |
| PH02Gene34597 | LOC_Os01g53650 | 0.265454223 | 0.44742105 | 0.593298467 | Purifying selection |
| PH02Gene23823 | LOC_Os01g61830 | 0.041353125 | 0.323951911 | 0.127652049 | Purifying selection |
| PH02Gene42371 | LOC_Os01g68860 | 0.050862358 | 0.24456864 | 0.207967621 | Purifying selection |
| PH02Gene10635 | LOC_Os01g07930 | 0.461413389 | 0.597478506 | 0.772267762 | Purifying selection |
| PH02Gene47743 | LOC_Os01g47750 | 0.148789191 | 0.581904367 | 0.255693546 | Purifying selection |
| PH02Gene04944 | LOC_Os01g61830 | 0.03765866 | 0.368380752 | 0.102227546 | Purifying selection |
| PH02Gene05739 | LOC_Os01g68860 | 0.048959652 | 0.238159144 | 0.205575362 | Purifying selection |
| PH02Gene29104 | LOC_Os01g07920 | 0.293998265 | 0.693836956 | 0.423728172 | Purifying selection |
| PH02Gene14940 | LOC_Os01g09620 | 0.079528016 | 0.373378821 | 0.212995519 | Purifying selection |
| PH02Gene43572 | LOC_Os01g15350 | 0.240110383 | 0.835999135 | 0.287213674 | Purifying selection |
| PH02Gene26949 | LOC_Os01g42970 | 0.139125907 | 0.320617385 | 0.433931264 | Purifying selection |
| PH02Gene32013 | LOC_Os01g45730 | 0.072648989 | 0.237749565 | 0.305569388 | Purifying selection |
| PH02Gene39677 | LOC_Os01g39100 | 0.224498174 | 0.451520943 | 0.497204342 | Purifying selection |
| PH02Gene08203 | LOC_Os01g09620 | 0.149623811 | 0.468073681 | 0.319658671 | Purifying selection |
| PH02Gene08811 | LOC_Os01g14870 | 0.113257513 | 0.23988198 | 0.472138477 | Purifying selection |
| PH02Gene40124 | LOC_Os01g15350 | 0.093379261 | 0.339802513 | 0.274804503 | Purifying selection |
| PH02Gene22259 | LOC_Os01g45730 | 0.215081792 | 0.377077758 | 0.570391085 | Purifying selection |
| PH02Gene40104 | LOC_Os01g09620 | 0.142497889 | 0.486849289 | 0.292694047 | Purifying selection |
| PH02Gene04182 | LOC_Os01g45730 | 0.237997797 | 0.375279425 | 0.634188238 | Purifying selection |
| PH02Gene04254 | LOC_Os01g47750 | 0.167749951 | 0.876127479 | 0.191467515 | Purifying selection |
| PH02Gene24845 | LOC_Os01g53650 | 0.33117699 | 0.716633584 | 0.462128761 | Purifying selection |
| PH02Gene22705 | LOC_Os10g36810 | 0.349722417 | 0.878877251 | 0.397919523 | Purifying selection |
| PH02Gene36671 | LOC_Os10g36810 | 0.221321596 | 0.560284187 | 0.395016673 | Purifying selection |
| PH02Gene27190 | LOC_Os10g25220 | 0.209789312 | 0.469665602 | 0.446678043 | Purifying selection |
| PH02Gene30888 | LOC_Os11g28270 | 0.048003953 | 0.33319585 | 0.144071281 | Purifying selection |
| PH02Gene34953 | LOC_Os11g28270 | 0.065738036 | 0.384297405 | 0.171060317 | Purifying selection |
| PH02Gene27671 | LOC_Os12g33090 | 0.066129112 | 0.386671181 | 0.17102157 | Purifying selection |
| PH02Gene26317 | LOC_Os12g18120 | 0.088620992 | 0.496818961 | 0.17837683 | Purifying selection |
| PH02Gene43485 | LOC_Os12g33090 | 0.065824101 | 0.389277575 | 0.169092969 | Purifying selection |
| PH02Gene03339 | LOC_Os02g45480 | 0.035938137 | 0.324964458 | 0.110590977 | Purifying selection |
| PH02Gene28052 | LOC_Os02g58440 | 0.146164092 | 0.430720114 | 0.339348193 | Purifying selection |
| PH02Gene10304 | LOC_Os02g10080 | 0.039062527 | 0.295646827 | 0.132125642 | Purifying selection |
| PH02Gene34123 | LOC_Os02g10080 | 0.133968177 | 0.513191097 | 0.261049301 | Purifying selection |
| PH02Gene06226 | LOC_Os02g35150 | 0.121524549 | 0.433744078 | 0.280175696 | Purifying selection |
| PH02Gene01488 | LOC_Os02g45480 | 0.057496706 | 0.364097724 | 0.157915587 | Purifying selection |
| PH02Gene27920 | LOC_Os02g58440 | 0.13832744 | 0.373578607 | 0.370276662 | Purifying selection |
| PH02Gene42383 | LOC_Os02g10080 | 0.104729596 | 0.703182388 | 0.148936602 | Purifying selection |
| PH02Gene33725 | LOC_Os03g49170 | 0.038823991 | 0.345236582 | 0.112456192 | Purifying selection |
| PH02Gene33170 | LOC_Os03g61110 | 0.086523027 | 0.326710496 | 0.264830875 | Purifying selection |
| PH02Gene22705 | LOC_Os03g02160 | 0.239839638 | 0.452871419 | 0.529597648 | Purifying selection |
| PH02Gene19288 | LOC_Os03g21140 | 0.101709997 | 0.416800419 | 0.244025659 | Purifying selection |
| PH02Gene12814 | LOC_Os03g21140 | 0.138670009 | 0.67121017 | 0.206597002 | Purifying selection |
| PH02Gene18259 | LOC_Os03g49170 | 0.03261718 | 0.408477505 | 0.079850614 | Purifying selection |
| PH02Gene49957 | LOC_Os03g21140 | 0.103424772 | 0.435072491 | 0.237718482 | Purifying selection |
| PH02Gene17992 | LOC_Os03g21140 | 0.145692681 | 0.619088774 | 0.23533407 | Purifying selection |
| PH02Gene03339 | LOC_Os04g48375 | 0.396066171 | 0.741656062 | 0.53402944 | Purifying selection |
| PH02Gene39245 | LOC_Os04g01480 | 0.096820368 | 0.419282621 | 0.230919105 | Purifying selection |
| PH02Gene44888 | LOC_Os04g32340 | 0.085444083 | 0.319238983 | 0.267649277 | Purifying selection |
| PH02Gene05204 | LOC_Os04g35800 | 0.123630054 | 0.317582959 | 0.389284281 | Purifying selection |
| PH02Gene20573 | LOC_Os04g57010 | 0.054111353 | 0.371199652 | 0.145774257 | Purifying selection |
| PH02Gene43143 | LOC_Os04g57600 | 0.125422167 | 0.431219509 | 0.290854574 | Purifying selection |
| PH02Gene16079 | LOC_Os04g32340 | 0.081461791 | 0.366176079 | 0.222466175 | Purifying selection |
| PH02Gene36785 | LOC_Os04g35800 | 0.111085593 | 0.313397824 | 0.354455535 | Purifying selection |
| PH02Gene00402 | LOC_Os04g56750 | 0.123176134 | 0.392804693 | 0.313581117 | Purifying selection |
| PH02Gene00385 | LOC_Os04g57010 | 0.054767481 | 0.439739081 | 0.124545403 | Purifying selection |
| PH02Gene00351 | LOC_Os04g57600 | 0.107231524 | 0.373923076 | 0.286774288 | Purifying selection |
| PH02Gene09684 | LOC_Os04g02730 | 1.249894717 | NaN | NaN |  |
| PH02Gene01488 | LOC_Os04g48375 | 0.426628601 | 0.718451326 | 0.593816986 | Purifying selection |
| PH02Gene47743 | LOC_Os05g48960 | 0.08947632 | 0.763287854 | 0.117224871 | Purifying selection |
| PH02Gene34597 | LOC_Os05g45020 | 0.248937496 | 0.609201007 | 0.408629489 | Purifying selection |
| PH02Gene45826 | LOC_Os05g45020 | 0.284223557 | 0.789709756 | 0.359908884 | Purifying selection |
| PH02Gene32013 | LOC_Os05g50080 | 0.238700723 | 0.418029038 | 0.571014692 | Purifying selection |
| PH02Gene14940 | LOC_Os05g10670 | 0.146549562 | 0.498963028 | 0.293708258 | Purifying selection |
| PH02Gene04361 | LOC_Os05g03760 | 0.078469296 | 0.377933553 | 0.207627228 | Purifying selection |
| PH02Gene08203 | LOC_Os05g10670 | 0.080298299 | 0.352309056 | 0.227920054 | Purifying selection |
| PH02Gene08040 | LOC_Os05g45020 | 0.073137695 | 0.342699955 | 0.213416121 | Purifying selection |
| PH02Gene22259 | LOC_Os05g50080 | 0.15121107 | 0.265665602 | 0.569178203 | Purifying selection |
| PH02Gene24845 | LOC_Os05g45020 | 0.071863995 | 0.3026346 | 0.237461267 | Purifying selection |
| PH02Gene04254 | LOC_Os05g48960 | 0.02941174 | 0.382462475 | 0.076900982 | Purifying selection |
| PH02Gene04182 | LOC_Os05g50080 | 0.134941832 | 0.280578171 | 0.480942019 | Purifying selection |
| PH02Gene25228 | LOC_Os05g03760 | 0.066373494 | 0.377295866 | 0.175918955 | Purifying selection |
| PH02Gene40104 | LOC_Os05g10670 | 0.064633272 | 0.401476778 | 0.160988819 | Purifying selection |
| PH02Gene36946 | LOC_Os06g46400 | 0.139692057 | 0.665876545 | 0.209786721 | Purifying selection |
| PH02Gene34666 | LOC_Os06g46890 | 0.294847612 | 0.709703673 | 0.415451721 | Purifying selection |
| PH02Gene10304 | LOC_Os06g41384 | 0.128646629 | 0.64468727 | 0.19954889 | Purifying selection |
| PH02Gene16813 | LOC_Os06g21390 | 0.779293318 | 1.804081859 | 0.431961174 | Purifying selection |
| PH02Gene02119 | LOC_Os06g46890 | 0.290530769 | 0.711271942 | 0.408466512 | Purifying selection |
| PH02Gene34123 | LOC_Os06g41384 | 0.224282206 | 0.977231959 | 0.229507645 | Purifying selection |
| PH02Gene19939 | LOC_Os06g07350 | 0.280622204 | 1.305783282 | 0.21490718 | Purifying selection |
| PH02Gene17257 | LOC_Os06g07350 | 0.028501444 | 0.381570615 | 0.074695071 | Purifying selection |
| PH02Gene29764 | LOC_Os06g49080 | 0.121576893 | 0.445335419 | 0.273000728 | Purifying selection |
| PH02Gene13668 | LOC_Os06g43120 | 0.185157765 | 0.391540616 | 0.472895423 | Purifying selection |
| PH02Gene27145 | LOC_Os06g46400 | 0.059124381 | 0.457095291 | 0.129348042 | Purifying selection |
| PH02Gene42261 | LOC_Os06g21390 | 0.061797862 | 0.266392478 | 0.231980506 | Purifying selection |
| PH02Gene12713 | LOC_Os06g07350 | 0.034203303 | 0.42746711 | 0.080013883 | Purifying selection |
| PH02Gene44958 | LOC_Os06g46890 | 0.165358208 | 0.37593676 | 0.439856449 | Purifying selection |
| PH02Gene42383 | LOC_Os06g41384 | 0.076155645 | 0.363252663 | 0.209649239 | Purifying selection |
| PH02Gene11220 | LOC_Os06g43120 | 0.752659397 | NaN | NaN |  |
| PH02Gene18149 | LOC_Os06g21390 | 0.075162194 | 0.241190647 | 0.311629805 | Purifying selection |
| PH02Gene06968 | LOC_Os07g38090 | 0.06567125 | 0.458788983 | 0.143140425 | Purifying selection |
| PH02Gene19288 | LOC_Os07g48410 | 0.116864763 | 0.537924243 | 0.21725134 | Purifying selection |
| PH02Gene12814 | LOC_Os07g48410 | 0.049142363 | 0.367055455 | 0.133882665 | Purifying selection |
| PH02Gene48688 | LOC_Os07g39440 | 0.607980776 | NaN | NaN |  |
| PH02Gene49957 | LOC_Os07g48410 | 0.113707668 | 0.51626079 | 0.220252381 | Purifying selection |
| PH02Gene42765 | LOC_Os07g38090 | 0.062316248 | 0.478213571 | 0.130310496 | Purifying selection |
| PH02Gene13318 | LOC_Os07g39440 | 0.239447157 | 0.428404304 | 0.55892799 | Purifying selection |
| PH02Gene17992 | LOC_Os07g48410 | 0.048024593 | 0.386972448 | 0.124103392 | Purifying selection |
| PH02Gene02576 | LOC_Os08g06330 | 0.113747397 | 0.65818392 | 0.172820079 | Purifying selection |
| PH02Gene15731 | LOC_Os08g04170 | 0.14126771 | 0.347973325 | 0.405972815 | Purifying selection |
| PH02Gene46793 | LOC_Os08g06330 | 0.05078561 | 0.477375609 | 0.106385013 | Purifying selection |
| PH02Gene04626 | LOC_Os09g31482 | 0.082229487 | 0.537352928 | 0.153026964 | Purifying selection |
| PH02Gene05151 | LOC_Os09g36090 | 0.183995611 | 0.508309002 | 0.361975905 | Purifying selection |
| PH02Gene12613 | LOC_Os09g31482 | 0.046676363 | 0.485807427 | 0.09607997 | Purifying selection |
| PH02Gene47633 | LOC_Os09g36090 | 0.202204579 | 0.528704561 | 0.382452874 | Purifying selection |
