# Supplementary material for: Identification of CCCH Zinc Finger Proteins Family in Moso Bamboo (Phyllostachys edulis), and PeC3H74 Confers Drought Tolerance to Transgenic Plants
Source: Front Plant Sci. 2020 Nov 9;11:579255. doi: 10.3389/fpls.2020.579255 (PMC7680867; doi:10.3389/fpls.2020.579255)
Supplement: Supplementary Table 6 — Ka/Ks value for duplicate CCCH genes between moso bamboo and maize. [file Table_6.DOC]

**Table S6. Ka/Ks value for duplicate CCCH genes between moso bamboo and maize**

| PeC3H Gene ID | OsC3H Gene ID | Ka | Ks | Ka/Ks | Selection pressure |
| --- | --- | --- | --- | --- | --- |
| PH02Gene06968 | Zm00001d006619_T001 | 0.090815206 | 0.571605039 | 0.158877545 | Purifying selection |
| PH02Gene06968 | Zm00001d021831_T001 | 0.085903391 | 0.502399279 | 0.170986293 | Purifying selection |
| PH02Gene26317 | Zm00001d023636_T001 | 0.127729781 | 0.666725226 | 0.191577843 | Purifying selection |
| PH02Gene27671 | Zm00001d023927_T001 | 0.079370921 | 0.560711378 | 0.141553969 | Purifying selection |
| PH02Gene27671 | Zm00001d041702_T001 | 0.078696648 | 0.530462716 | 0.14835472 | Purifying selection |
| PH02Gene43485 | Zm00001d023927_T001 | 0.079926286 | 0.606207421 | 0.131846433 | Purifying selection |
| PH02Gene43485 | Zm00001d041702_T001 | 0.080960544 | 0.533997121 | 0.151612323 | Purifying selection |
| PH02Gene30888 | Zm00001d004651_T001 | 0.06334422 | 0.42181724 | 0.150169822 | Purifying selection |
| PH02Gene30888 | Zm00001d049223_T001 | 0.063427281 | 0.469513395 | 0.135091525 | Purifying selection |
| PH02Gene22177 | Zm00001d044260_T001 | 0.314848673 | 0.695527287 | 0.452676234 | Purifying selection |
| PH02Gene23823 | Zm00001d042965_T001 | 0.048768663 | 0.436441159 | 0.111741667 | Purifying selection |
| PH02Gene42371 | Zm00001d042363_T001 | 0.114665777 | 0.490290712 | 0.233873035 | Purifying selection |
| PH02Gene34597 | Zm00001d038772_T001 | 0.263886234 | 0.677550092 | 0.38947118 | Purifying selection |
| PH02Gene19983 | Zm00001d011355_T001 | 0.305008001 | 0.785702587 | 0.388197781 | Purifying selection |
| PH02Gene10635 | Zm00001d008735_T001 | 0.397996015 | 0.643984674 | 0.618020942 | Purifying selection |
| PH02Gene42371 | Zm00001d012049_T001 | 0.058383059 | 0.399288133 | 0.146217867 | Purifying selection |
| PH02Gene33170 | Zm00001d034710_T001 | 0.162969981 | 0.484731177 | 0.336206931 | Purifying selection |
| PH02Gene22705 | Zm00001d027334_T001 | 0.194515363 | 0.543132055 | 0.358136407 | Purifying selection |
| PH02Gene33725 | Zm00001d033610_T001 | 0.050399925 | 0.527461244 | 0.095551902 | Purifying selection |
| PH02Gene19288 | Zm00001d029010_T001 | 0.098299502 | 0.499040933 | 0.196976833 | Purifying selection |
| PH02Gene33725 | Zm00001d013497_T001 | 0.05148889 | 0.513680372 | 0.100235268 | Purifying selection |
| PH02Gene22705 | Zm00001d048510_T002 | 0.187195203 | 0.533788467 | 0.350691733 | Purifying selection |
| PH02Gene04944 | Zm00001d042965_T001 | 0.045019434 | 0.472566783 | 0.095265761 | Purifying selection |
| PH02Gene05739 | Zm00001d042363_T001 | 0.11891454 | 0.489126985 | 0.243115886 | Purifying selection |
| PH02Gene14940 | Zm00001d039495_T001 | 0.107265943 | 0.480602777 | 0.223190436 | Purifying selection |
| PH02Gene32013 | Zm00001d044074_T001 | 0.106732564 | 0.232265954 | 0.459527374 | Purifying selection |
| PH02Gene39677 | Zm00001d044260_T001 | 0.290463716 | 0.712345916 | 0.407756554 | Purifying selection |
| PH02Gene43572 | Zm00001d040301_T001 | 0.27330075 | 0.963686931 | 0.283599104 | Purifying selection |
| PH02Gene14940 | Zm00001d037769_T001 | 0.165615854 | 0.54050389 | 0.306410106 | Purifying selection |
| PH02Gene32013 | Zm00001d039101_T001 | 0.292217746 | 0.492875463 | 0.592883533 | Purifying selection |
| PH02Gene45826 | Zm00001d038772_T001 | 0.324246983 | 0.899843559 | 0.360337061 | Purifying selection |
| PH02Gene05739 | Zm00001d012049_T001 | 0.056178765 | 0.478371679 | 0.117437482 | Purifying selection |
| PH02Gene14940 | Zm00001d008812_T001 | 0.114190937 | 0.443902269 | 0.257243419 | Purifying selection |
| PH02Gene29104 | Zm00001d008735_T001 | 0.655665474 | 1.056959634 | 0.620331612 | Purifying selection |
| PH02Gene32013 | Zm00001d011470_T001 | 0.142887713 | 0.271030152 | 0.527202277 | Purifying selection |
| PH02Gene26949 | Zm00001d011355_T001 | 0.3113545 | 0.712388683 | 0.437057055 | Purifying selection |
| PH02Gene10304 | Zm00001d053682_T001 | 0.14445884 | 0.375517801 | 0.384692389 | Purifying selection |
| PH02Gene34666 | Zm00001d053841_T001 | 0.090238972 | 0.50540433 | 0.178548078 | Purifying selection |
| PH02Gene28052 | Zm00001d051838_T001 | 0.236715683 | 0.614199451 | 0.385405233 | Purifying selection |
| PH02Gene03339 | Zm00001d017595_T001 | 0.101122491 | 0.612988818 | 0.164966289 | Purifying selection |
| PH02Gene18357 | Zm00001d018360_T001 | 0.083661006 | 0.393291388 | 0.212720157 | Purifying selection |
| PH02Gene16813 | Zm00001d017853_T001 | 0.700118396 | 2.065972815 | 0.33888074 | Purifying selection |
| PH02Gene10304 | Zm00001d046740_T001 | 0.220882706 | 0.979418778 | 0.225524271 | Purifying selection |
| PH02Gene08203 | Zm00001d039495_T001 | 0.156370823 | 0.528635955 | 0.295800583 | Purifying selection |
| PH02Gene02576 | Zm00001d049516_T001 | 0.174249758 | 1.030011326 | 0.169172662 | Purifying selection |
| PH02Gene08203 | Zm00001d037769_T001 | 0.101469015 | 0.37437883 | 0.271032992 | Purifying selection |
| PH02Gene04361 | Zm00001d035455_T001 | 0.072863979 | 0.399096694 | 0.182572245 | Purifying selection |
| PH02Gene04626 | Zm00001d020954_T001 | 0.112022934 | 0.731609702 | 0.153118437 | Purifying selection |
| PH02Gene08203 | Zm00001d010380_T001 | 0.096856926 | 0.384679355 | 0.25178613 | Purifying selection |
| PH02Gene04361 | Zm00001d010956_T001 | 0.117534777 | 0.36353091 | 0.323314398 | Purifying selection |
| PH02Gene12814 | Zm00001d029010_T001 | 0.139896067 | 0.635127694 | 0.220264474 | Purifying selection |
| PH02Gene12814 | Zm00001d022427_T001 | 0.098616703 | 0.428995838 | 0.229877995 | Purifying selection |
| PH02Gene34953 | Zm00001d004651_T001 | 0.090161029 | 0.435799503 | 0.206886489 | Purifying selection |
| PH02Gene34953 | Zm00001d049223_T001 | 0.086333847 | 0.463656635 | 0.186202117 | Purifying selection |
| PH02Gene34123 | Zm00001d053682_T001 | 0.234943668 | 0.575073675 | 0.408545337 | Purifying selection |
| PH02Gene02119 | Zm00001d053841_T001 | 0.092359236 | 0.493573707 | 0.187123492 | Purifying selection |
| PH02Gene34123 | Zm00001d036796_T001 | 0.208877831 | 0.991242212 | 0.210723301 | Purifying selection |
| PH02Gene18259 | Zm00001d033610_T001 | 0.051328654 | 0.552520592 | 0.092899079 | Purifying selection |
| PH02Gene49957 | Zm00001d029010_T001 | 0.106864031 | 0.465215306 | 0.22970876 | Purifying selection |
| PH02Gene48688 | Zm00001d048569_T001 | 0.298161623 | 0.645534702 | 0.461883183 | Purifying selection |
| PH02Gene15731 | Zm00001d024703_T001 | 0.177705552 | 0.549615116 | 0.323327264 | Purifying selection |
| PH02Gene46793 | Zm00001d049516_T001 | 0.228418639 | 1.131451719 | 0.201881031 | Purifying selection |
| PH02Gene05204 | Zm00001d025450_T001 | 0.174075359 | 0.632375417 | 0.275272178 | Purifying selection |
| PH02Gene43143 | Zm00001d026587_T001 | 0.194526482 | 0.493826796 | 0.393916417 | Purifying selection |
| PH02Gene20573 | Zm00001d026543_T001 | 0.11906653 | 0.642142063 | 0.185420854 | Purifying selection |
| PH02Gene39245 | Zm00001d024928_T001 | 0.142919391 | 0.600347207 | 0.238061224 | Purifying selection |
| PH02Gene20573 | Zm00001d001933_T001 | 0.092592966 | 0.708265713 | 0.130731962 | Purifying selection |
| PH02Gene44888 | Zm00001d003922_T001 | 0.135034546 | 0.396383091 | 0.340666766 | Purifying selection |
| PH02Gene39245 | Zm00001d004541_T001 | 0.13035588 | 0.564845699 | 0.230781398 | Purifying selection |
| PH02Gene36785 | Zm00001d025450_T001 | 0.384024332 | 1.011458195 | 0.379673953 | Purifying selection |
| PH02Gene00351 | Zm00001d026587_T001 | 0.184907846 | 0.477204339 | 0.387481485 | Purifying selection |
| PH02Gene00402 | Zm00001d026536_T001 | 0.191552449 | 0.663440575 | 0.288725858 | Purifying selection |
| PH02Gene00385 | Zm00001d026543_T001 | 0.118437261 | 0.659917283 | 0.179472888 | Purifying selection |
| PH02Gene00402 | Zm00001d001952_T001 | 0.156999846 | 0.603475396 | 0.260159481 | Purifying selection |
| PH02Gene00385 | Zm00001d001933_T001 | 0.091225334 | 0.709788977 | 0.128524585 | Purifying selection |
| PH02Gene16079 | Zm00001d003922_T001 | 0.131126743 | 0.4222646 | 0.310532171 | Purifying selection |
| PH02Gene27920 | Zm00001d051838_T001 | 0.233166349 | 0.594389009 | 0.392279039 | Purifying selection |
| PH02Gene01488 | Zm00001d017595_T001 | 0.100551951 | 0.631531205 | 0.159219292 | Purifying selection |
| PH02Gene12613 | Zm00001d020954_T001 | 0.105828626 | 0.771261962 | 0.137214891 | Purifying selection |
| PH02Gene42765 | Zm00001d006619_T001 | 0.093115635 | 0.58532867 | 0.159082649 | Purifying selection |
| PH02Gene17992 | Zm00001d022427_T001 | 0.09564124 | 0.419059123 | 0.228228511 | Purifying selection |
| PH02Gene13318 | Zm00001d021945_T001 | 0.516312873 | 0.676497091 | 0.763215216 | Purifying selection |
| PH02Gene42765 | Zm00001d021831_T001 | 0.087214383 | 0.505344065 | 0.172584165 | Purifying selection |
| PH02Gene36671 | Zm00001d032470_T001 | 0.248770793 | 0.577933466 | 0.430448844 | Purifying selection |
| PH02Gene40124 | Zm00001d040301_T001 | 0.151176246 | 0.545391411 | 0.277188535 | Purifying selection |
| PH02Gene29764 | Zm00001d014705_T001 | 0.139728515 | 0.445071008 | 0.313946568 | Purifying selection |
| PH02Gene27145 | Zm00001d014378_T001 | 0.057913732 | 0.561694521 | 0.103105388 | Purifying selection |
| PH02Gene27145 | Zm00001d036594_T001 | 0.074682899 | 0.683330114 | 0.109292562 | Purifying selection |
| PH02Gene08811 | Zm00001d008356_T001 | 0.237750565 | 0.513801495 | 0.462728442 | Purifying selection |
| PH02Gene40127 | Zm00001d008326_T001 | 0.134326142 | 0.521309604 | 0.257670568 | Purifying selection |
| PH02Gene40124 | Zm00001d008322_T001 | 0.245503493 | 0.881452974 | 0.278521374 | Purifying selection |
| PH02Gene13668 | Zm00001d046630_T001 | 0.187266373 | 0.58880547 | 0.318044553 | Purifying selection |
| PH02Gene17257 | Zm00001d045183_T001 | 0.049538518 | 0.491097332 | 0.100873116 | Purifying selection |
| PH02Gene22259 | Zm00001d044074_T001 | 0.212516318 | 0.375003975 | 0.566704173 | Purifying selection |
| PH02Gene22259 | Zm00001d039101_T001 | 0.220359254 | 0.360009789 | 0.612092397 | Purifying selection |
| PH02Gene08040 | Zm00001d038772_T001 | 0.110220554 | 0.325325485 | 0.338800858 | Purifying selection |
| PH02Gene44958 | Zm00001d053841_T001 | 0.337540513 | 1.038701056 | 0.32496406 | Purifying selection |
| PH02Gene42383 | Zm00001d053682_T001 | 0.182598821 | 0.742037472 | 0.246077628 | Purifying selection |
| PH02Gene44958 | Zm00001d014472_T001 | 0.229837826 | 0.563681035 | 0.407744471 | Purifying selection |
| PH02Gene42383 | Zm00001d036796_T001 | 0.113924675 | 0.448729146 | 0.253882939 | Purifying selection |
| PH02Gene44958 | Zm00001d036560_T001 | 0.203827931 | 0.530217183 | 0.384423473 | Purifying selection |
| PH02Gene12713 | Zm00001d045183_T001 | 0.054496215 | 0.522527845 | 0.104293418 | Purifying selection |
| PH02Gene11220 | Zm00001d046630_T001 | 1.480748059 | NaN | NaN |  |
| PH02Gene42383 | Zm00001d046740_T001 | 0.192391124 | 0.556891391 | 0.345473332 | Purifying selection |
| PH02Gene04182 | Zm00001d044074_T001 | 0.237960276 | 0.364638374 | 0.652592522 | Purifying selection |
| PH02Gene40104 | Zm00001d039495_T001 | 0.146497432 | 0.504708355 | 0.290261555 | Purifying selection |
| PH02Gene04254 | Zm00001d039014_T001 | 0.062285741 | 0.499939611 | 0.124586529 | Purifying selection |
| PH02Gene04182 | Zm00001d039101_T001 | 0.214946603 | 0.325257558 | 0.660850447 | Purifying selection |
| PH02Gene24845 | Zm00001d038772_T001 | 0.10598515 | 0.510370121 | 0.207663312 | Purifying selection |
| PH02Gene40104 | Zm00001d037769_T001 | 0.084463018 | 0.394219807 | 0.214253615 | Purifying selection |
| PH02Gene25228 | Zm00001d035455_T001 | 0.081826265 | 0.382571773 | 0.213884741 | Purifying selection |
| PH02Gene04254 | Zm00001d009570_T001 | 0.07039536 | 0.555342236 | 0.126760321 | Purifying selection |
| PH02Gene24845 | Zm00001d010800_T001 | 0.091577023 | 0.37532983 | 0.243990792 | Purifying selection |
| PH02Gene40104 | Zm00001d010380_T001 | 0.083049763 | 0.375036782 | 0.221444315 | Purifying selection |
| PH02Gene25228 | Zm00001d010956_T001 | 0.093511916 | 0.342672053 | 0.272890408 | Purifying selection |
| PH02Gene04182 | Zm00001d011470_T001 | 0.274138189 | 0.396316542 | 0.691715233 | Purifying selection |
| PH02Gene40104 | Zm00001d008812_T001 | 0.138846842 | 0.508969862 | 0.272799732 | Purifying selection |
