# Supplementary material for: Identification of CCCH Zinc Finger Proteins Family in Moso Bamboo (Phyllostachys edulis), and PeC3H74 Confers Drought Tolerance to Transgenic Plants
Source: Front Plant Sci. 2020 Nov 9;11:579255. doi: 10.3389/fpls.2020.579255 (PMC7680867; doi:10.3389/fpls.2020.579255)
Supplement: Supplementary Table 7 — Kinds and numbers of known stress-related elements in the upstream regions of CCCH genes in moso bamboo. [file Table_7.DOC]

Table S7. Kinds and numbers of known stress-related elements in the upstream regions of CCCH genes in moso bamboo.

| **Element** | **ABRE** | **CGTCA motif / TGACG motif** | **GARE motif / P box / TATC-box** | **Element** | **ABRE** | | **CGTCA motif / TGACG motif** | | **GARE motif / P box / TATC-box** | |  |
| --- | --- | --- | --- | --- | --- | --- | --- | --- | --- | --- | --- |
| Function | ABA | MeJA | GA | Function | ABA | | MeJA | | GA | |  |
| PeC3H1 | 0 | 0 | 1 | PeC3H61 | 7 | | 3 | | 0 | |  |
| PeC3H2 | 2 | 3 | 1 | PeC3H62 | 2 | | 4 | | 2 | |  |
| PeC3H3 | 6 | 3 | 0 | PeC3H63 | 7 | | 0 | | 3 | |  |
| PeC3H4 | 4 | 2 | 0 | PeC3H64 | 6 | | 3 | | 1 | |  |
| PeC3H5 | 2 | 3 | 1 | PeC3H65 | 6 | | 5 | | 1 | |  |
| PeC3H6 | 9 | 2 | 0 | PeC3H66 | 8 | | 3 | | 0 | |  |
| PeC3H7 | 7 | 4 | 0 | PeC3H67 | 5 | | 1 | | 1 | |  |
| PeC3H8 | 1 | 1 | 0 | PeC3H68 | 4 | | 0 | | 2 | |  |
| PeC3H9 | 8 | 6 | 1 | PeC3H69 | 1 | | 1 | | 0 | |  |
| PeC3H10 | 3 | 9 | 2 | PeC3H70 | 1 | | 3 | | 0 | |  |
| PeC3H11 | 2 | 1 | 1 | PeC3H71 | 3 | | 1 | | 0 | |  |
| PeC3H12 | 2 | 1 | 3 | PeC3H72 | 4 | | 2 | | 3 | |  |
| PeC3H13 | 2 | 1 | 0 | PeC3H73 | 2 | | 3 | | 1 | |  |
| PeC3H14 | 4 | 3 | 1 | PeC3H74 | 1 | | 3 | | 3 | |  |
| PeC3H15 | 3 | 3 | 0 | PeC3H75 | 0 | | 4 | | 0 | |  |
| PeC3H16 | 0 | 0 | 0 | PeC3H76 | 2 | | 5 | | 1 | |  |
| PeC3H17 | 2 | 0 | 0 | PeC3H77 | 0 | | 1 | | 0 | |  |
| PeC3H18 | 0 | 3 | 0 | PeC3H78 | 1 | | 4 | | 2 | |  |
| PeC3H19 | 6 | 3 | 1 | PeC3H79 | 2 | | 3 | | 0 | |  |
| PeC3H20 | 0 | 7 | 0 | PeC3H80 | 0 | | 3 | | 2 | |  |
| PeC3H21 | 2 | 0 | 1 | PeC3H81 | 1 | | 2 | | 1 | |  |
| PeC3H22 | 3 | 3 | 0 | PeC3H82 | 3 | | 0 | | 0 | |  |
| PeC3H23 | 10 | 2 | 3 | PeC3H83 | 5 | | 1 | | 2 | |  |
| PeC3H24 | 6 | 0 | 1 | PeC3H84 | 11 | | 8 | | 3 | |  |
| PeC3H25 | 1 | 1 | 2 | PeC3H85 | 5 | | 4 | | 0 | |  |
| PeC3H26 | 5 | 2 | 0 | PeC3H86 | 0 | | 0 | | 1 | |  |
| PeC3H27 | 5 | 2 | 0 | PeC3H87 | 0 | | 4 | | 2 | |  |
| PeC3H28 | 4 | 3 | 0 | PeC3H88 | 3 | | 3 | | 0 | |  |
| PeC3H29 | 6 | 2 | 2 | PeC3H89 | 7 | | 3 | | 2 | |  |
| PeC3H30 | 3 | 0 | 0 | PeC3H90 | 5 | | 0 | | 0 | |  |
| PeC3H31 | 0 | 2 | 2 | PeC3H91 | 7 | | 2 | | 0 | |  |
| PeC3H32 | 0 | 1 | 1 | PeC3H92 | 1 | | 1 | | 0 | |  |
| PeC3H33 | 2 | 2 | 2 | PeC3H93 | 3 | | 2 | | 1 | |  |
| PeC3H34 | 3 | 3 | 0 | PeC3H94 | 0 | | 1 | | 0 | |  |
| PeC3H35 | 3 | 2 | 0 | PeC3H95 | 10 | | 7 | | 0 | |  |
| PeC3H36 | 4 | 4 | 1 | PeC3H96 | 4 | | 3 | | 5 | |  |
| PeC3H37 | 1 | 5 | 2 | PeC3H97 | 5 | | 1 | | 1 | |  |
| PeC3H38 | 2 | 1 | 0 | PeC3H98 | 4 | | 1 | | 0 | |  |
| PeC3H39 | 1 | 2 | 0 | PeC3H99 | 3 | | 5 | | 1 | |  |
| PeC3H40 | 8 | 4 | 3 | PeC3H100 | 15 | | 0 | | 0 | |  |
| PeC3H41 | 0 | 1 | 0 | PeC3H101 | 3 | | 2 | | 0 | |  |
| PeC3H42 | 19 | 5 | 0 | PeC3H102 | 1 | | 5 | | 0 | |  |
| PeC3H43 | 2 | 2 | 1 | PeC3H103 | 4 | | 3 | | 2 | |  |
| PeC3H44 | 3 | 3 | 2 | PeC3H104 | 5 | | 3 | | 0 | |  |
| PeC3H45 | 2 | 0 | 5 | PeC3H105 | 2 | | 2 | | 0 | |  |
| PeC3H46 | 0 | 1 | 0 | PeC3H106 | 5 | | 2 | | 2 | |  |
| PeC3H47 | 4 | 1 | 1 | PeC3H107 | 4 | | 4 | | 0 | |  |
| PeC3H48 | 10 | 5 | 0 | PeC3H108 | 2 | | 5 | | 2 | |  |
| PeC3H49 | 2 | 5 | 0 | PeC3H109 | 14 | | 2 | | 3 | |  |
| PeC3H50 | 1 | 0 | 0 | PeC3H110 | 13 | | 6 | | 2 | |  |
| PeC3H51 | 1 | 4 | 0 | PeC3H111 | 3 | | 1 | | 1 | |  |
| PeC3H52 | 0 | 6 | 0 | PeC3H112 | 4 | | 3 | | 0 | |  |
| PeC3H53 | 7 | 6 | 0 | PeC3H113 | 2 | | 0 | | 1 | |  |
| PeC3H54 | 1 | 0 | 0 | PeC3H114 | 1 | | 6 | | 0 | |  |
| PeC3H55 | 4 | 0 | 1 | PeC3H115 | 1 | | 1 | | 1 | |  |
| PeC3H56 | 6 | 6 | 1 | PeC3H116 | 0 | | 1 | | 1 | |  |
| PeC3H57 | 4 | 1 | 1 | PeC3H117 | 0 | | 4 | | 2 | |  |
| PeC3H58 | 5 | 0 | 0 | PeC3H118 | 6 | | 1 | | 0 | |  |
| PeC3H59 | 1 | 2 | 0 | PeC3H119 | 8 | | 3 | | 1 | |  |
| PeC3H60 | 3 | 2 | 1 |  | |  | |  | | 1 | |
