# Supplementary material for: Identification of CCCH Zinc Finger Proteins Family in Moso Bamboo (Phyllostachys edulis), and PeC3H74 Confers Drought Tolerance to Transgenic Plants
Source: Front Plant Sci. 2020 Nov 9;11:579255. doi: 10.3389/fpls.2020.579255 (PMC7680867; doi:10.3389/fpls.2020.579255)
Supplement: Supplementary Table 8 — The 12 gene primer sequences. [file Table_8.DOC]

Table.S8 The 12 gene primer sequences.

| **Name** |  | **Primer sequences** |
| --- | --- | --- |
| *PeC3H2* | Forward | ATCAACTCCATCCTCCAATCCC |
|  | Reverse | CGCTGTCACTTGGCGTCCT |
| *PeC3H7* | Forward | ACCAGCCGTGAAGACTCGC |
|  | Reverse | TTGGCAGGGGCTCAAAGTAA |
| *PeC3H11* | Forward | CTGCGATGATGACGACAAAGTT |
|  | Reverse | CAAAGGGGCACTCAGTCCAG |
| *PeC3H20* | Forward | GAGGAGGAGCCCGTGGAGA |
|  | Reverse | GGAGACGGTGGCTTCTTTGC |
| *PeC3H21* | Forward | CGACGGCAGCGAAGGACT |
|  | Reverse | AGCACGCAGCGTTCAAAGA |
| *PeC3H26* | Forward | CGATGGGTAAGGTTGACTGGG |
|  | Reverse | ATTCGGCTCCGCTTCTGC |
| *PeC3H34* | Forward | CTTCCTCAACCTTCGGTGCTT |
|  | Reverse | GTTTGGCCCCAGTCATTCG |
| *PeC3H56* | Forward | GCCCAACTGGCTGGAAATC |
|  | Reverse | GGCATCCCTCCTCGGTGTA |
| *PeC3H74* | Forward | CAGCCAATGCCCAACCCT |
|  | Reverse | CTCCACCCCTCCCGATGA |
| *PeC3H99* | Forward | TCCACCCCGAGGGCTACTA |
|  | Reverse | GATGAGGCCGAGATCCAATG |
| *PeC3H100* | Forward | GCACGACGAGCAGTCCAAGG |
|  | Reverse | GCAGTAGCCAGTCATCTCCCAC |
| *PeC3H110* | Forward | ATCCCACCGCAGCTCCTC |
|  | Reverse | AACTCGTCGTCCGCCTCC |
| *TIP41* | Forward | AAAATCATTGTAGGCCATTGTCG |
|  | Reverse | ACTAAATTAAGCCAGCGGGAGTG |
